# Rheologically Engineered 3D-Printed Highly Loaded Magneto-Dielectric Absorbers for Device-Level Electromagnetic Compatibility

**DOI:** 10.1007/s40820-026-02312-7

**Published:** 2026-07-30

**Authors:** Yuheng Jiang, Zihao Chen, Xiao Sun, Haotian Li, Jinlong Xie, Yueting Li, Xiaolei Nie, Feng Lan, Yaxin Zhang, Qiye Wen

**Affiliations:** 1 School of Electronic Science and Engineering, University of Electronic Science and Technology of China, Chengdu, 611731 People’s Republic of China; 2 Shenzhen Institute for Advanced Study, University of Electronic Science and Technology of China, Shenzhen, 518110 People’s Republic of China; 3 Engineering Center of Integrated Optoelectronic & Radio Meta-Chips, Chengdu, 611731 People’s Republic of China

**Keywords:** Direct ink writing (DIW) 3D printing, Rheologically engineered, Highly loaded composite inks, Magneto-dielectric composite, Ultra-broadband absorption

## Abstract

Highly loaded magneto-dielectric composite systems are promising for achieving strong electromagnetic loss and structural design flexibility, yet their processability and geometric controllability remain extremely challenging. Herein, a rheologically engineered direct ink writing (DIW) 3D printing strategy is developed. Graphene (Gr) is introduced to reconstruct the particle-loading network, which synergistically improves the ink’s yield behavior, shear-thinning property, structural recovery, and a magnetic–dielectric synergistic loss system is constructed with carbonyl iron powder (CIP). The critical CIP content (~ 84.06 wt%) is determined by the yield model, and a tunable Gr/CIP (GC) composite ink is obtained. The correlation among rheological response, geometric fidelity, and temporal stability is established, enabling high-fidelity 3D-printed gradient honeycomb structures. The rationally designed 3D-printed GC honeycomb (GCH) absorber achieves an effective absorption bandwidth of 18 GHz–4 THz, with an RL_min_ of − 84.30 dB at a thickness of 2.6 mm. When integrated into device-level terahertz reconfigurable intelligent surfaces (RIS), the GCH absorber contributes to a ~ 3.3 dBi main lobe gain enhancement, 1.9–3.3 dB sidelobe suppression, and a ~ 58% reduction in reflection beamwidth, showing excellent electromagnetic compatibility performance for communication, imaging, and radar. This work offers a practical strategy for the structural fabrication and device integration of high-load magneto-dielectric synergistic absorbers.

**Supplementary Information:**

The online version contains supplementary material available at 10.1007/s40820-026-02312-7.

## Highlights

- A rheologically engineered approach enables the 3D printing of high-loading (~84.06 wt%) magneto-dielectric composite inks, with quantitative composition–rheology–printability relationships established.
- 3D-printed GC honeycomb (GCH) absorber achieves ultra-broadband absorption covering 18 GHz–4 THz, with reflection loss (RL) ≤ − 10 dB and RL_min_ of − 84.30 dB in the terahertz band.
- The monolithic integration of the GCH absorber with a terahertz reconfigurable intelligent surface delivers a 3.3 dBi increase in main lobe gain and ~58% reduction in specular reflection beamwidth.

**Supplementary Information:**

The online version contains supplementary material available at 10.1007/s40820-026-02312-7.

## Graphical abstract

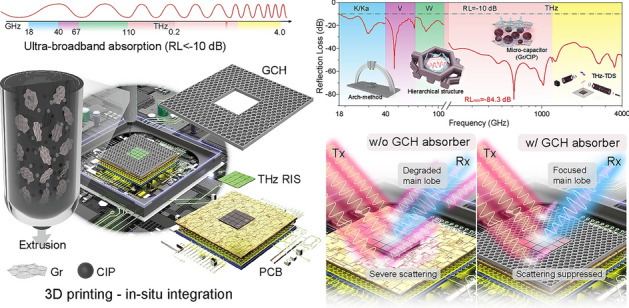

## Introduction

The evolution of wireless communication, radar detection, and radio astronomy into higher-frequency bands has expanded electromagnetic system operations from traditional microwaves to millimeter-wave (MMW) and terahertz (THz) bands [1–3]. High-frequency electromagnetic waves (EMWs) offer significant advantages in spectrum resources, spatial resolution, and imaging accuracy, underpinning cutting-edge technologies such as 6G communication, short-range high-speed interconnection, and deep space observation [4]. However, existing microwave modules and MMW/THz spectrum need to coexist harmoniously, and electromagnetic compatibility (EMC) issues between them need to be resolved [5, 6]. Therefore, it is urgent to develop cross-band, ultra-wideband electromagnetic absorbing materials (EAMs) that can cover MMW–THz to effectively suppress stray EMW energy. Concurrently, device and system integration trends necessitate ideal EAMs to exhibit multifunctional properties, including ultra-thinness, flexibility, and conformal, heterogeneous structures [7]. This makes it difficult for traditional EAMs that rely on thickness compensation or are designed for a single frequency band to meet the application requirements of the new generation of high-frequency electromagnetic systems [8].

Most current research focuses on a single frequency domain in low-frequency microwave or MMW-THz bands, while EAMs that can achieve continuous coverage from GHz to THz bands still face significant technical bottlenecks [9, 10]. To overcome these limitations, composition/microstructure engineering has been widely explored to tailor electromagnetic parameters, impedance matching, and attenuation capability [11–13]. Among these strategies, magnetic–dielectric synergistic EAMs have attracted increasing attention in recent years [12–14]. In such systems, soft magnetic fillers represented by carbonyl iron powder (CIP) usually show significant magnetic loss response in the GHz band [15, 16]. Combining them with dielectric materials can achieve effective absorption in higher-frequency bands [17, 18]; Huang et al. [19] achieved an EAB of 30 GHz in 2–40 GHz by optimizing the CIP/MWCNT composition. However, relying solely on composition control often makes it difficult to simultaneously consider impedance matching and absorption intensity [20]. Structural design offers a feasible solution to these challenges. Through engineered gradients, porous, or topological structures, it optimizes impedance matching and extends electromagnetic wave propagation paths [21]; Wang et al. [22] proposed slotted all-cement dielectric superstructure, which achieved an EAB of over 34.6 GHz and an average reflection loss (RL_ave_) of − 23.4 dB. However, existing structural forming approaches still rely on traditional processes such as the template method, hot pressing, and mold forming, which can only produce simple absorbing structures. These methods can hardly satisfy the demands of devices, components, and microsystems in the MMW/THz bands, because their characteristic dimensions have approached the submillimeter scale [23, 24].

Direct ink writing (DIW) 3D printing, which uses continuous extrusion of non-Newtonian fluids to construct complex, millimeter/submillimeter-scale heterogeneous structures, thus presents a novel manufacturing path for multi-component composites and device integration [25, 26]. Compared with mold-based processing for simple repetitive geometries, DIW 3D printing offers mold-free programmability for constructing gradient, heterogeneous, and conformal architectures for localized or modular functional-layer integration [27]. However, the central premise for DIW 3D printing compatibility involves delicately balancing ink functionalization and processing performance [28]. Achieving electromagnetic functionality requires ensuring precise printable rheological characteristics, including significant shear thinning for extrusion, rapid elastic network recovery post-deposition, and a sufficient yield modulus (G') to prevent spreading and creep [29]. This balance is particularly precarious in EAM design: Low filler loading inhibits electromagnetic loss, necessitating reliance on thick structures for broadband absorption [30]. Conversely, the high loading (> 50 wt%) required for improved loss severely disrupts this balance [31, 32]. High-density spherical magnetic fillers, notably CIP, exacerbate this issue due to their severe settling tendency, weak load-bearing networks, and time-dependent structural rearrangement [33]. These factors decrease extrusion consistency and deteriorate geometric fidelity, significantly compressing the DIW printable window. Crucially, there remains a lack of a quantitative rheological engineering framework that connects material composition, rheological response, and DIW printing geometric fidelity, meaning synergistic optimization remains heavily reliant on empirical iteration [34]. This limitation mainly arises from the complex coupling between particle packing, interparticle interactions, and polymer network dynamics in highly filled inks, making it difficult to establish predictive relationships between rheological parameters and printing fidelity [35].

To address these challenges, we present a rheology-driven ink design strategy based on a representative multi-dimensional Gr/CIP composite system, in which high-density spherical CIP serves as a magnetic loss filler with typical high-loading processability challenges, while 2D Gr acts as both an electrical loss provider and a rheological regulation unit. Benefiting from its sheet-like morphology, Gr reconstructs the CIP particle-supporting network while introducing conductive loss channels, thereby improving yield support, shear thinning, structural recovery, and electromagnetic attenuation without relying on chemical crosslinking or low-loading formulations. We constructed a GC ink system with varying Gr/CIP ratios to uncover the intrinsic relationships governing rheological response, printability, and geometric fidelity. The resulting 3D-printed GC honeycomb (GCH) absorber achieved ultra-broadband absorption (RL ≤ − 10 dB) spanning 18 GHz–4 THz. Notably, at a thickness of only 2.6 mm, it exhibited a minimum reflection loss (RL_min_) of − 84.30 dB. Furthermore, integrating the 3D-printed GCH absorber around a THz reconfigurable intelligent surface (RIS) substantially enhanced main lobe gain and sidelobe suppression while mitigating specular reflection width. It also demonstrated significant scattering suppression in MMW imaging/radar applications. These findings demonstrate that rheological manipulation provides a feasible path for the structured fabrication and device-level integration of highly loaded magneto-dielectric synergistic EAMs.

## Experimental Section

### Materials

Graphene (Gr) was purchased from Suzhou Carbon-Feng Graphene Technology Co., Ltd., China. Purity 99.9%, thickness range: 3.4–8.0 nm, sheet size: 5–20 *μ*m. Carbonyl iron powder (CIP) was purchased from Jiangyou Hebao Nanomaterials Co., Ltd., purity 99.9%, particle size range: 1–5 *μ*m. Polydimethylsiloxane (PDMS, BD-6184) elastomer was purchased from Baoerde (Hangzhou Baldsil New Material Technology Co., Ltd., China) as a two-component kit consisting of a base polymer (component A) and a curing agent (component B). All reagents were used in their original packaging without any further purification.

### Preparation of Print-Ready GC Ink

To prevent Gr from adsorbing and deactivating the platinum (Pt) catalyst in the PDMS curing system, a premixing strategy was employed in the ink preparation. First, PDMS component A and component B were mixed at room temperature in a mass ratio of 10:1 and mechanically stirred for 5 min to ensure thorough dispersion and coating of the catalyst by PDMS molecules. This process created an effective physical barrier within the system, thereby reducing the direct adsorption of free catalyst onto the graphene surface. Subsequently, CIP was added to the system and stirred for 5 min to achieve initial dispersion. Gr was then added and stirred for another 5 min to form the initial composite ink. The specific formulation of each ink is shown in Table S1. To further improve the dispersion of the filler, the ink was subjected to shear dispersion treatment using a three-roll mill (ZYTR-50, Shenzhen Zhongyi Technology Co., Ltd., Shenzhen, China), with a grinding gap of 30 *μ*m, and the process was repeated 3–5 times. Finally, the ink was transferred to a vacuum stirring and defoaming device (BT-300, Shenzhen Boveton Precision Technology Co., Ltd., Shenzhen, China) for defoaming treatment, resulting in a uniformly dispersed, bubble-free functional ink for subsequent rheological testing and DIW printing experiments.

### Printing and Curing of GCH Absorber

The prepared ink is loaded into the dispensing barrel and processed through a three-axis synchronized tabletop dispensing printing system (TS-200BN, Shenzhen Tensun Precision Equipment, China), with DIW 3D printing executed via a pneumatic extrusion module. The printing process follows a pre-designed CAD path, with key parameters (nozzle diameter, extrusion pressure, printing speed, and layer height) detailed in Table S2. Upon completion, the printed structures undergo 4-h thermal curing at 60 °C to achieve crosslinking of the PDMS matrix, ultimately yielding GCH absorber with stable 3D structures.

### Material Characterization and Testing

All rheological tests were conducted on a stress-controlled rotational rheometer (MCR 302e, Anton Paar GmbH, Graz, Austria). Flow behavior was obtained through flow sweep tests with shear rates ranging from 0.01 to 100 s^−1^. The viscoelastic properties of the ink were characterized by oscillatory stress scanning at a frequency of 1 Hz and a stress range of 0.1–10,000 Pa. The thixotropic recovery behavior of the ink was evaluated using a three-stage shear program: first, loading at a low shear rate (0.01 s^−1^) for 180 s to simulate the resting state; followed by high shear rates (1.0 or 10.0 s^−1^) for 180 s to simulate the extrusion process; and finally, returning to 0.01 s^−1^ and holding for 180 s to characterize the structural reconfiguration capability. Frequency sweep measurements were conducted within the linear viscoelastic region (LVR) over an angular frequency (*ω*) range of 0.5–100 rad s^−1^. All the above rheological tests were carried out at 25 °C. The 2D geometric fidelity was evaluated using image analysis methods. The top view images of 3D-printed mesh structures (Axio Observer 7, Carl Zeiss Microscopy GmbH, Jena, Germany) underwent sequential image preprocessing, binary segmentation, and morphological correction to obtain computable 2D pore masks. Pore geometric features were extracted based on pixel counting and connected component analysis, and the 2D geometric fidelity was quantitatively assessed using the printability index (Pr) and Dice overlap coefficient. Here, Pr approaching 1 indicates pore geometry close to ideal square. Pr < 1 typically corresponds to pore rounding, while Pr > 1 signifies pore boundary distortion. The Dice coefficient reflects the consistency between printed structures and CAD designs in terms of area overlap, with values closer to 1 indicating higher overall geometric consistency.

The surface and cross-sectional microstructures of the 3D structures were observed using a field emission scanning electron microscope (FE-SEM, Gemini SEM 300, Carl Zeiss, Germany) at an acceleration voltage of 10 kV. Elemental distribution was analyzed by energy-dispersive X-ray spectroscopy (EDS mapping). Cross-sectional samples were prepared by brittle fracture after 3 min of liquid nitrogen cryogenic treatment. All samples underwent 30-s gold spraying prior to observation to enhance conductivity. Fourier transform infrared spectroscopy (FTIR) spectra were measured using a Bruker-VERTEX 80v. X-ray photoelectron spectroscopy (XPS) tests were performed with a Thermo Fisher K-Alpha instrument.

The terahertz (THz) absorption characteristics are measured using a commercial all-fiber system (Fico-TM, Zomega) to obtain THz time-domain spectra (THz–TDS) within the effective spectral range of 0.2–4.5 THz at a repetition frequency of 1 kHz. By performing a fast Fourier transform on the time-domain signal, the wave amplitude and phase are determined, enabling accurate extraction of the material’s reflectivity in the terahertz band. The reflection loss (RL) of the sample is calculated using the following formula:

1 $${\mathrm{RL}} \left( {dB} \right) = - 20\log_{10} (|E_{{\mathrm{i}}} |/|E_{{\mathrm{r}}} |)$$

Here, ${E}_{i}$ and ${E}_{\mathrm{r}}$ represent the terahertz pulse amplitudes reflected by the test sample and reference aluminum plate, respectively. The RL of samples in the microwave/MMW bands was mainly measured using the free-space arch method with arch test stands corresponding to each frequency band. For the W band (75–110 GHz) measurement, a vector network analyzer (VNA, AV3672C, Ceyear Technologies Co., Ltd., China) equipped with a compatible millimeter-wave extension module (AV3640A, Ceyear Technologies Co., Ltd., China) and a standard horn antenna pair was used. All measurements were conducted in a microwave anechoic chamber to minimize environmental reflection interference.

Thermal conductivity of the circular samples (12.5 mm in diameter, 2 mm in thickness) was measured at 70 °C using a laser flash analyzer (LFA 467, NETZSCH, Germany). A digital constant-temperature heating platform (Model ET-200, ETOOL, Shenzhen Bangqi Chuangyuan Technology Co., Ltd., China) was used for sample heating at a controlled temperature of 100 °C. An infrared thermal imager (Model HIKMICRO E10, Hangzhou Microimage Sensing Technology Co., Ltd., Hangzhou, China) was employed to record the surface temperature and thermal evolution of the samples during heating and cooling processes.

## Results and Discussion

### Rheological Properties of GC Ink

Typically, DIW 3D printing filaments do not undergo rapid solidification after deposition, with the structural stability of the printed object entirely dependent on the ink’s inherent elastic network support. Consequently, the ink must maintain continuous and stable extrusion flow within the nozzle while rapidly regaining sufficient mechanical support post-deposition to preserve the structure’s shape [36]. This fundamentally relies on the ink’s rheological properties, including shear thinning, thixotropic recovery, and high static viscosity and structural strength under low shear or static conditions [36].

For highly loaded functional composite inks, this rheological equilibrium is further constrained by the upper limit of filler volume fraction. When the filler volume fraction (*φ*) approaches the maximum packing volume fraction (*φ*_m_), enhanced particle interactions cause viscosity (*η*) and yield stress (*τ*_y_) to exhibit nonlinear steep increases, which may readily lead to nozzle clogging [37, 38]. Therefore, determining the *φ*_*m*_ and yield response of each filler system is a critical prerequisite for developing high solid content, printable inks. First, the yield behavior of a single filler system is analyzed (Fig. S1a, b): Both pure Gr and pure CIP inks demonstrate nonlinear growth characteristics of *τ*_y_ with increasing *φ*. This behavior can be described by the yield stress-volume fraction model proposed by Heymann et al. [39]:

2 $$\tau_{{\mathrm{y}}} = \tau^{*} \left[ {\left( {1 - \frac{\varphi }{{\varphi_{{\mathrm{m}}} }}} \right)^{ - 2} - 1} \right]$$

**Fig. 1:**
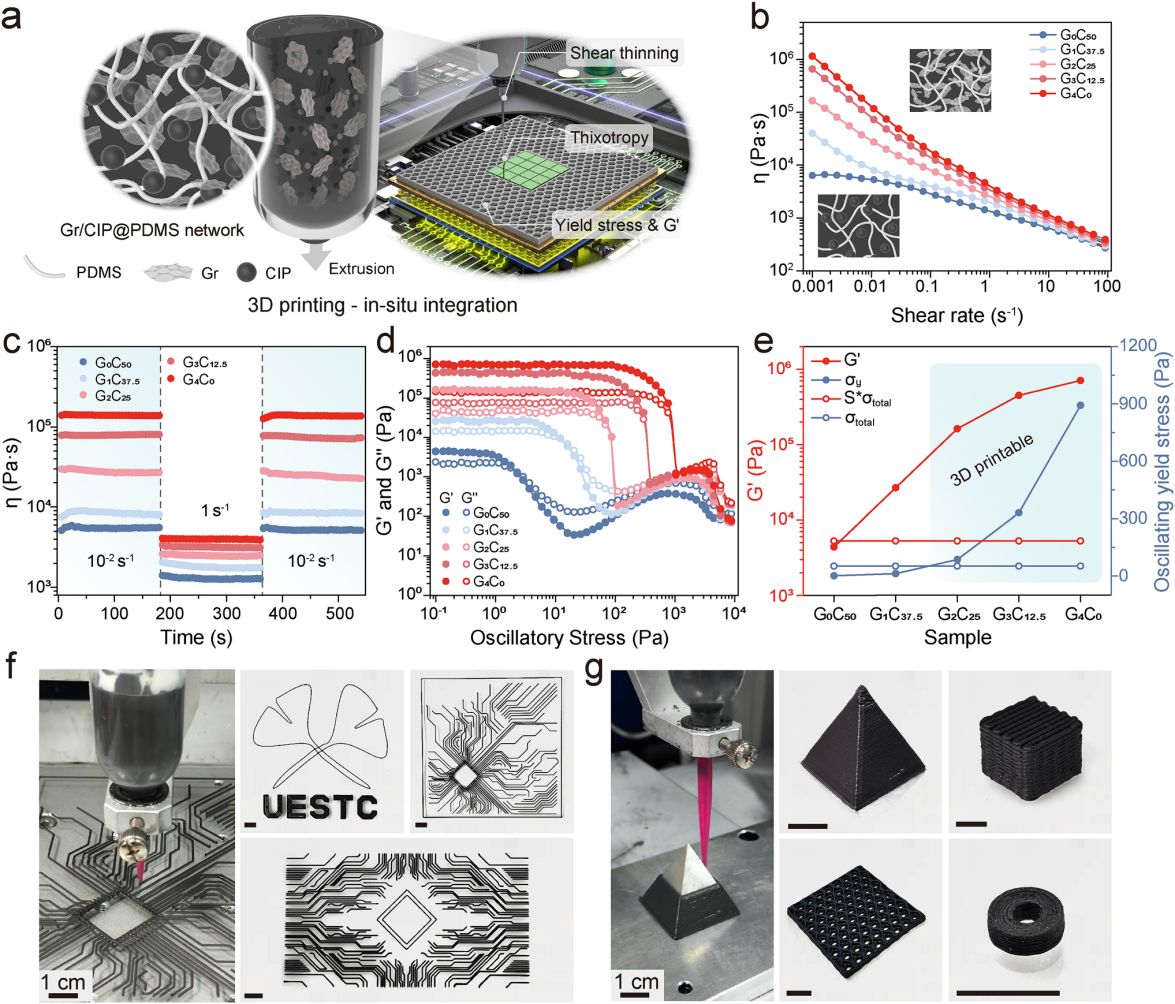
Rheological behavior of GC series inks and feasibility evaluation of DIW printing. **a** Microscopic network of Gr/CIP-PDMS composite inks and in situ 3D printing on electromagnetic devices. **b** *η*-$\dot{\gamma }$ curves of GC inks with different Gr/CIP ratios. **c** *η*-time curves with alternating low $\dot{\gamma }$ (0.01 s^−1^) and high $\dot{\gamma }$ (1 s^−1^) applications. **d** G' and G" as functions of oscillatory stress. **e** *G*' in the linear viscoelastic region and *σ*y at the flow point, along with corresponding S *σ*total and *σ*total. **f**–**g** Representative DIW printing examples of GC inks, scale: 1 cm

Here, τ^*^ denotes the characteristic stress scale associated with matrix properties, while *φ*_m_ represents the maximum effective bulk volume fraction achievable in the system. The fitting yielded *φ*_m, Gr_ = 0.152 and *φ*_m, CIP_ = 0.396, which are 28.90 and 84.06 wt%, respectively. This characterizes the different packing/load capacity limits of the two sheet-like and spherical packings under high loads. Gr significantly enhances *τ*_y_ at much lower *φ* values than CIP, attributed to the elastic network formed by Gr flakes, whereas CIP’s yield enhancement primarily depends on increased particle contact numbers, with relatively limited network carrying capacity and reversibility [40, 41]. Using *φ*_m,Gr_ and *φ*_m,CIP_ as reference limits for composite formulations, G_4_C_0_ (*φ*_Gr_ = 0.138, 26.6 wt%) and G_0_C_50_ (*φ*_CIP_ = 0.361, 81.97 wt%) were selected as the endpoints for formulation scanning while maintaining processing margins. Subsequently, a series of GC inks were constructed by gradually replacing CIP with Gr to investigate the effects of the Gr/CIP ratio on rheological response and DIW printability. Figure S1c demonstrates that under low shear conditions (0.01 s^−1^), the *η* increases nonlinearly with the Gr ratio, preliminarily revealing Gr’s efficient thickening effect. Figure 1 further presents the rheological response of GC inks and validation of DIW feasibility. Figure 1a demonstrates the microstructure of the Gr/CIP-PDMS composite ink and its in situ 3D printing integration on electromagnetic devices. Flow scan results (Fig. 1b) reveal that all GC inks exhibit typical shear-thinning behavior, with both the shear viscosity (*η*) and the degree of shear-thinning increasing proportionally as the Gr content rises, which effectively reduces extrusion resistance and ensures post-deposition conformal integrity. In Fig. S2a, the shear stress (*τ*) versus shear rate ($\dot{\gamma }$) curves are fitted using the Herschel–Bulkley (H-B) model [42]:

3 $$\tau = \tau_{{\mathrm{y}}} + K\dot{\gamma }^{n}$$

Here, *τ*_y_ denotes the yield stress, *K* is the viscosity coefficient, and n represents the rheological index. The fitting results (Fig. S2b–e) demonstrate that both *τ*_y_ and *K* increase with rising Gr, while n slightly decreases and consistently remains < 1, further indicating that the system maintains good shear-thinning properties while enhancing static load-bearing capacity [40]. Furthermore, Fig. 1c investigates thixotropy by analyzing the *η* variation of the ink under alternating shear rates of 0.01 and 1 s^−1^. Under constant $\dot{\gamma }$, the ink exhibits similar steady-state viscosity contributions. When $\dot{\gamma }$ is switched, *η* responds rapidly: The low shear state at 0.01 s^−1^ mimics the ink’s pre-loading behavior, where *η* remains high. Upon increasing the shear rate to 1 s^−1^, *η* drops sharply, indicating partial disruption of the internal network and shear-thinning behavior, which facilitates smooth extrusion from the nozzle. Subsequently, as $\dot{\gamma }$ decreases again, *η* recovers rapidly, with a recovery rate exceeding 95% within 180 s, demonstrating the ink’s excellent reversible reconfiguration capability and ensuring shape stability after deposition [36]. Figure 1d investigates the viscoelastic properties of GC ink through low-frequency oscillatory stress scanning, plotting the storage modulus (*G*') and loss modulus (*G*"). Within the low-stress range, all samples exhibit linear viscoelastic behavior with *G*' > *G*", demonstrating solid-like characteristics. As stress increases, *G*' begins to decrease and reaches a flow point at *G*' = *G*", where the characteristic stress is defined as the oscillatory yield stress (*σ*_y_) [40]. Further stress increases disrupt the internal network structure of the ink, triggering solid–liquid transition and entering a viscous-dominated flow state. For extruded filament deposits, geometric instability is primarily driven by two forces: surface tension (*σ*_cap_)-induced spreading and self-weight (*σ*_*g*_) induced creep/collapse [28]. Thus, the external equivalent stress (*σ*_total_) acting on the filament during deposition can be expressed as [38]:

4 $$\sigma_{{{\mathrm{total}}}} = \sigma_{{{\mathrm{cap}}}} + \sigma_{{\mathrm{g}}} = \frac{\Gamma }{r} + \rho {\mathrm{gh}}$$

Here, *Γ* denotes the effective surface tension coefficient of the ink, *r* represents the characteristic radius of the deposited filaments, *ρ* is the ink density, *g* is the gravitational acceleration, and *h* is the characteristic deposition height [38, 43]. For the ink to rapidly cease flow and maintain its shape after deposition, the condition *σ*_y_ > *σ*_total_ must be satisfied [38, 44]. Additionally, to prevent significant elastic deformation and time-dependent creep within the linear viscoelastic region, the threshold *G*' > *S*·*σ*_total_ [45] is typically required, where *S* is an engineering empirical coefficient (generally ranging from 10 to 100. In this study, *S* = 100 is adopted as a conservative criterion to emphasize structural stability [45]). Figure 1e compares the values of *G*' and *σ*_y_ corresponding to the flow point in the linear viscoelastic region with the thresholds. Clearly, as the Gr content increases, both *G*' and *σ*_y_ exhibit upward trends, indicating the system progressively enters a rheological window that combines extrusibility and deposition stability. Frequency sweep results further reveal that Gr-containing GC inks exhibit much higher *G*' and *G*" values than PDMS and G_0_C_50_, with weaker frequency dependence (Fig. S3). This behavior suggests the formation of a more stable physical network and a slower relaxation process, which can be attributed to the restriction of PDMS chain relaxation and particle rearrangement by the multi-dimensional Gr/CIP network [46]. Cross-sectional SEM images further support this network reconstruction, showing that spherical CIP particles are spatially embedded among interconnected Gr sheets in G_2_C_25_, in contrast with the CIP-dominated G_0_C_50_ and Gr-dominated G_4_C_0_ structures (Fig. S4). Based on the rheological analysis, representative GC inks were tested for DIW printing (Fig. 1f, g). The results demonstrated successful formation of complex 2D paths and 3D stacked structures within the predicted rheological window, along with conformal printing on the surface of the metal pyramid. These results confirm the excellent feasibility of DIW printing with GC inks.

### Printing Performance of GC Ink

**Fig. 2:**
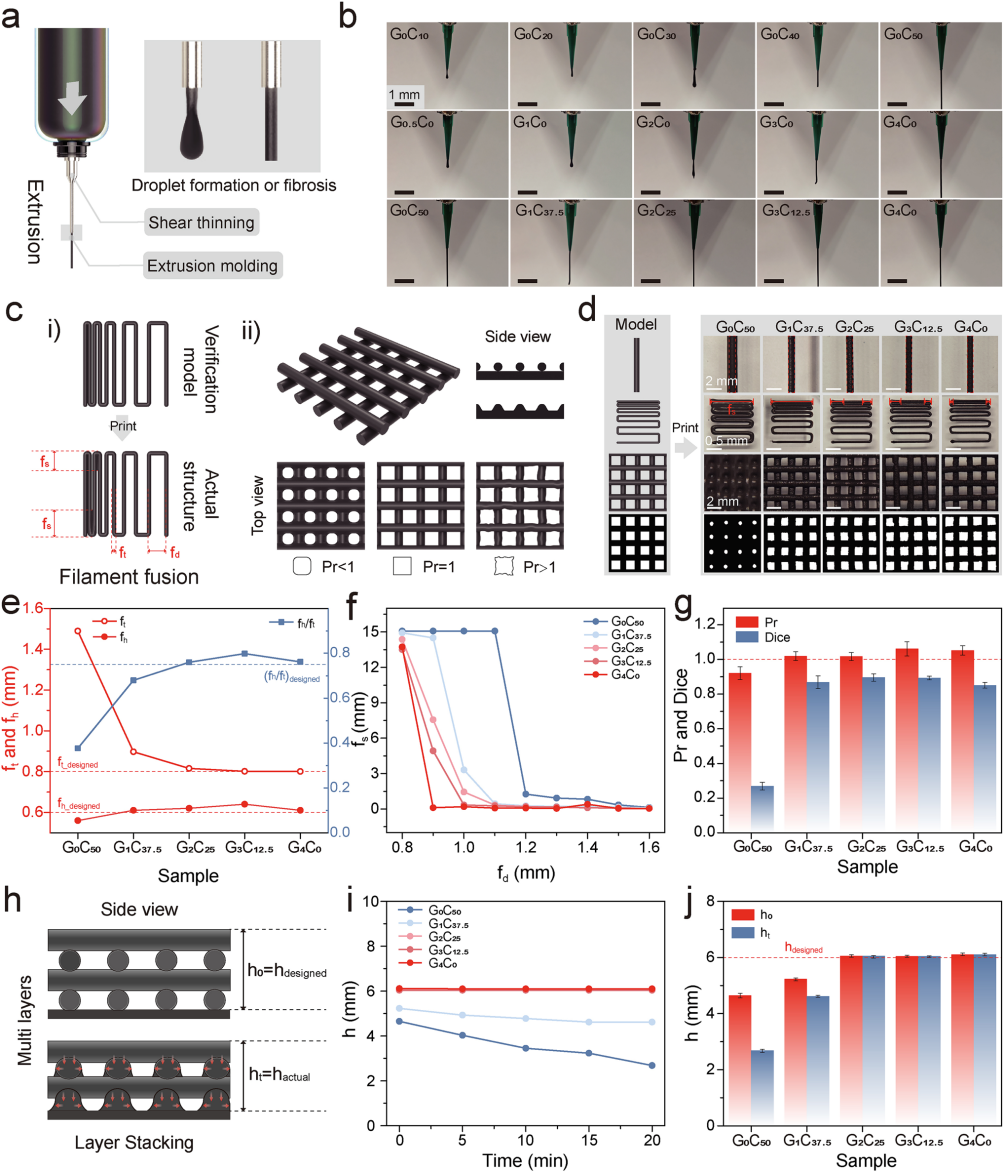
Print fidelity evaluation of GC series inks. **a** Schematic diagram of ink forming droplets or continuous filaments during extrusion. **b** Comparison of ink extrusion morphology (droplets/filaments), scale bar: 1 mm. **c** Schematic diagram of 2D fidelity evaluation criteria: i) meandering filament fusion test, ii) grid resolution test and aperture shape criteria. **d** Representative results of 2D geometry: comparison of CAD model and printed structure. **e** Statistics of *f*_*t*_, *f*_*h*_, and *f*_*h*_/*f*_*t*_. **f** *f*_*s*_–*f*_*d*_ relationship curves of different GC inks. **g** Pr and Dice of printed grid. **h** Schematic diagram of creep/collapse of multilayer stacked structure. **i** Evolution curve of lattice structure height with deposition time for different formulations. **j** *h*_0_ and *h*_*t*_ of lattice structure after 20 min of deposition

In Fig. 1, the rheological window required for stable extrusion and deposition of GC ink was established through rheological analysis, clarifying the printing feasibility under different Gr/CIP ratios. Therefore, Fig. 2 further evaluates the printing quality of GC ink from the perspective of geometric fidelity and verifies its intrinsic correlation with rheological response. Figure 2a shows the process of ink undergoing high shear, shear thinning, and forming droplets/continuous filaments in the nozzle during extrusion. The corresponding digital photograph is shown in Fig. 2b: Single filler inks (G_x_C_0_ or G_0_C_x_) mainly drip at low filler content. As the filler content increases, the structural strength of the system increases, and the extrusion morphology gradually changes from dripping to continuous filaments [47]. In contrast, GC composite inks can all achieve continuous extrusion and form filaments with uniform morphology, indicating that the multi-dimensional Gr/CIP synergistic network can provide sufficient cohesion and extrusion stability within a wide composition window [28, 41].

After filament deposition, the geometric fidelity of the x–y plane is mainly controlled by the time-dependent flow after deposition. When the ink yield stress and structural strength are insufficient to quickly suppress the flow, pore rounding and resolution loss are inevitable. To quantitatively characterize this fusion tendency, this paper uses a filament fusion test with meandering and stepped spacing (Fig. 2ci) [47]. In this test, as the filament spacing (*f*_d_) gradually decreases, the fusion region will occur and expand from the corner until local or even complete closure occurs, resulting in resolution loss in the x–y plane [29]. Figure 2cii further shows a schematic diagram of the mesh resolution test and evaluates the 2D geometric fidelity using the printability index (Pr) and Dice overlap coefficient [48]. Figure 2d shows the 3D model and representative printing results and quantifies the ability to retain geometric information after filament deposition by comparing the model with the actual printed structure. Figure 2e shows the filament width (*f*_*t*_), filament height (*f*_h_), and aspect ratio (*f*_h_/*f*_*t*_) of each formulation filament (design value is 2/3). With increasing Gr ink content, *f*_*t*_ gradually decreases from ~ 1.5 mm to close to the design filament width of ~ 0.8 mm, indicating effective suppression of lateral spreading. Simultaneously, *f*_*h*_ slightly increases but remains generally stable within the 0.55–0.65 mm range; *f*_h_/*f*_*t*_ gradually increases and approaches the design ratio (right axis of Fig. 2e). These results demonstrate that the introduction of Gr significantly reduces post-deposition lateral dimensional distortion and enhances 2D geometric accuracy. Figure 2f presents the *f*_*s*_–*f*_*d*_ relationship curve. G_0_C_50_ exhibits the strongest fusion tendency, showing a long fusion segment even at larger spacing (*f*_*d*_ > 1 mm) (*f*_*s*_ close to the upper limit of the range), indicating significant spreading and adhesion after deposition. As the Gr content increases, the *f*_*s*_ value shortens significantly and approaches 0 within a wider *f*_*d*_ range, indicating that the ink can form elastic support more quickly after deposition, enhancing the ability to maintain the boundaries of adjacent filaments [47]. This trend is consistent with the linewidth convergence results in Fig. 2e. Figure 2g further shows the Pr and Dice parameters of the printed mesh. Obviously, except for G_0_C_50_, the Pr values of the other formulations are close to 1 (approximately 1.0–1.06), indicating that the overall pore profile is close to an ideal square. The Pr value of G_0_C_50_ is significantly less than 1, corresponding to the geometric distortion caused by pore rounding and fusion [47]. Meanwhile, the Dice values of G_1_C_37.5_–G_4_C_0_ remain at a high level (approximately 0.85–0.90), indicating good consistency between the overall printed structure and the CAD model, while the Dice value of G_0_C_50_ is significantly low (~ 0.25), reflecting severe adhesion/missing parts, making it difficult to maintain the mesh topology [48, 49].

Besides 2D fidelity, the key challenge to the stability of 3D-printed structures comes from gravity-driven creep and interlayer collapse after deposition [28]. Figure 2h shows the lattice height loss during multilayer stacking. If time-dependent flow continues after ink deposition, the actual height ht will be lower than the designed height *h*_0_ [47]. Figure 2i records the evolution of lattice height over time. Combined with Fig. S5, the height of G_0_C_50_ continuously decreases and its geometric features gradually weaken within 0–20 min, showing significant creep and collapse. As the Gr content increases, the height decreases significantly, and G_2_C_25_–G_4_C_0_ remains basically stable, indicating that its deposited lattice can effectively resist gravity-driven time-dependent deformation. At *t* = 20 min (Fig. 2j), the *h*_*t*_ of G_2_C_25_–G_4_C_0_ is still close to *h*_0_, while G_0_C_50_ and G_1_C_37.5_ show significant height loss. The above results are consistent with the trends of filament fusion behavior (Fig. 2f) and 2D fidelity index (Fig. 2g), indicating that a certain Gr ratio can simultaneously improve the pore resolution in the x–y plane and the temporal stability of interlayer stacking in the z direction.

### Structural Characterization and Absorption Mechanism

**Fig. 3:**
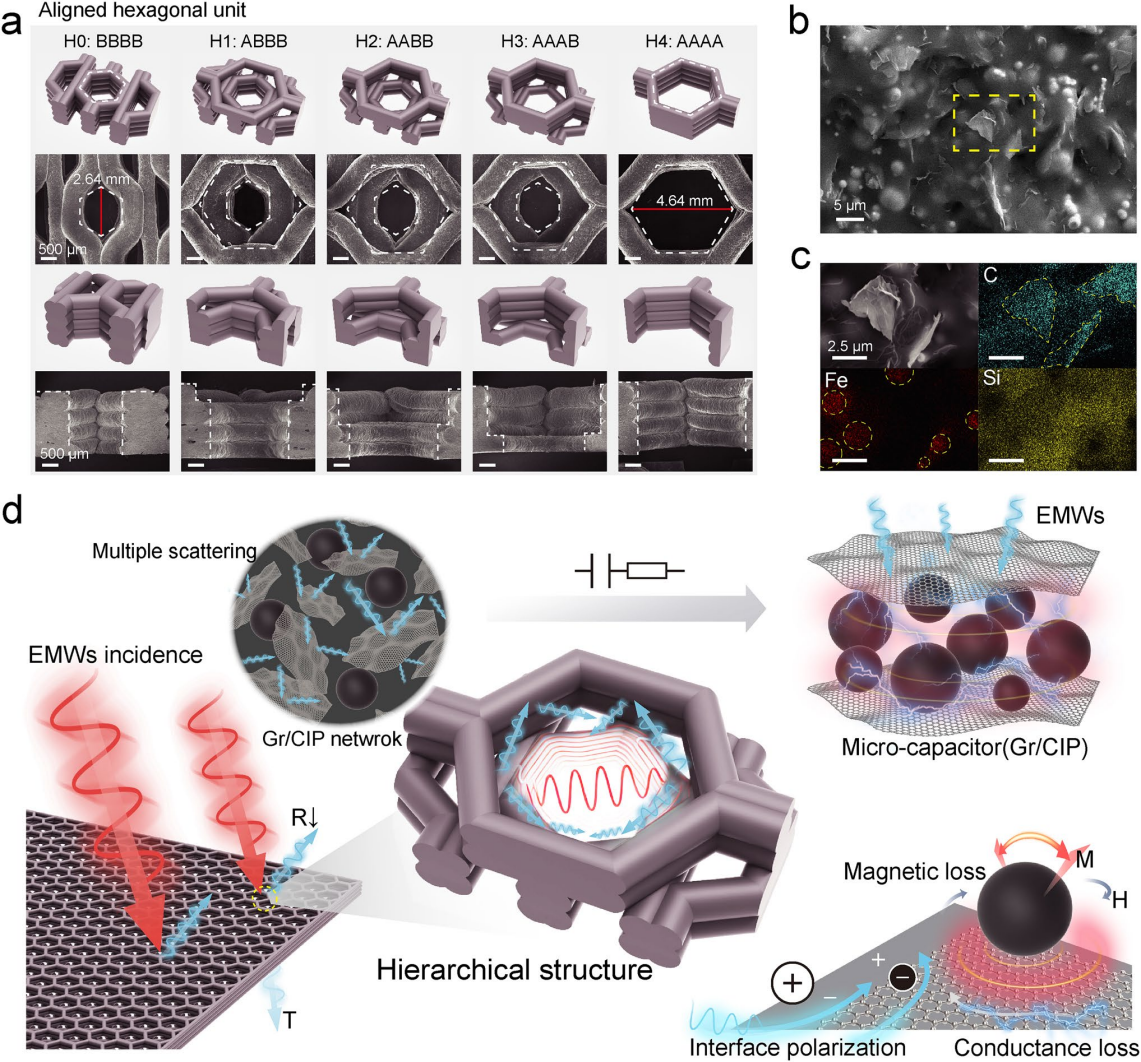
Morphological characterization and electromagnetic absorption mechanism of GCH absorber. **a** 3D model and SEM morphology of five gradient cell unit combinations constructed based on DIW technology. **b** SEM image of brittle cross section. **c** High-magnification SEM image and corresponding EDS mapping. **d** Schematic diagram of EMW absorption mechanism

The GCH absorber designed in this study is based on the electromagnetic propagation characteristics of the *W* band (75–110 GHz, wavelength coverage 2.7–4.0 mm) and terahertz band (0.2–1.2 THz, wavelength coverage 0.25–1.5 mm). Two representative cell aperture sizes were selected as basic units: a smaller aperture B (2.6 mm) to enhance the coupling and scattering of MMW/THz with the structure, and a larger aperture A (4.6 mm) to facilitate broadband impedance matching. Figure 3a shows the geometric design and actual printed morphology of five-unit combinations (H0–H4) of the GCH. By adjusting the combination of aperture units, A and B in the thickness direction (from BBBB to AAAA), a gradient evolution of the cell aperture from small to large was achieved. The first row shows a 3D model of the GCH structural unit, and the second row shows the corresponding SEM images, showing the process of the top layer aperture size gradually increasing from approximately 2.6–4.6 mm. Meanwhile, the continuous interlayer interface and uniform wall thickness of the printed layers indicate that GC ink has good forming stability and structural fidelity. Furthermore, the third row provides a schematic diagram of the cross-sectional morphology of each combination, highlighting the layer-by-layer stacking and geometric transition of the honeycomb wall along the thickness direction. As the proportion of A layer units increases, the honeycomb structure evolves from multilayer small pores to multilayer large pores. This geometric gradient along the thickness direction is conducive to introducing a smoother impedance transition in the propagation direction and enhancing the multiple scattering process of EMWs in the cavity [50]. The cross-sectional SEM image of the fourth row verifies the high fidelity of the interlayer stacking of the filaments in the thickness direction. The interfaces between each layer are continuous and the layer thickness is uniform, with no occurrence of collapse or delamination defects.

Figure 3b shows the SEM image of the micro-brittle fracture surface of the Gr/CIP-PDMS composite material. It can be observed that the CIP microspheres are uniformly dispersed in the continuous sheet network composed of Gr sheets. This multiphase composite system composed of Gr sheets–CIP magnetic spheres–PDMS matrix is conducive to the formation of many heterogeneous interfaces [51]. In addition, Fig. 3c shows the high-magnification SEM morphology and the corresponding EDS-Mapping. Among them, Fe mainly comes from CIP, C corresponds to the Gr sheet network, and Si comes from the PDMS matrix. Many CIP-PDMS-Gr heterostructures are formed, thus constructing a multi-dimensional, multi-interface composite network structure. To further reveal the effect of Gr content changes on the composite system, FTIR spectral analysis was performed on samples with different ratios (Fig. S6). It was revealed that the increase in Gr content caused the interfacial polarization in the system to change from disordered dipole relaxation to a dual mechanism of interfacial polarization and conductive loss [52]. XPS results (Fig. S7) show that with increasing Gr content, the C–C/C=C component in the C 1*s* spectrum is significantly enhanced, while the relative proportions of oxygen-containing carbon components O–C=O gradually decrease. This change is mainly attributed to the gradual increase in the surface contribution of high-purity graphitized Gr and its dilution effect on the oxygen-containing components in the CIP. Therefore, multilayer Gr gradually becomes the dominant component in the surface chemical composition of the composite system. In addition, the multilevel interfaces and sandwich structures formed between the sheets can generate stronger Maxwell–Wagner polarization under high-frequency excitation, which enhances energy dissipation [53].

Figure 3d analyzes the multi-mechanism synergistic absorption mechanism of 3D-printed GCH absorbers from the perspectives of macroscopic structure and microscopic composition. On the macroscopic scale, when EMWs are incident on the surface of the gradient honeycomb structure, more electromagnetic energy is coupled into the interior of the structure due to the equivalent impedance gradient formed by the gradual change of aperture (Fig. S8). In addition, the incident EMWs undergo multiple refractions and scatterings in the honeycomb cavity, and the propagation path is significantly extended, so they are gradually absorbed or attenuated during the transmission process [51]. On the microscopic composition, on the one hand, the large-diameter Gr sheet provides a conductive path for carriers to migrate and scatter in the high-frequency electric field, generating stable conductive losses [54]. At the same time, CIP microspheres introduce hysteresis loss and natural resonance loss under the action of an alternating magnetic field [55] and form a magnetic–dielectric synergistic attenuation mechanism with the electrical loss process of Gr. On the other hand, the highly conductive Gr sheets form a large number of micro-capacitor structures, while the introduction of CIP microspheres increases the spacing between Gr sheet layers, resulting in significant interfacial polarization and capacitive polarization losses under the action of high-frequency alternating electromagnetic fields [56, 57]. Furthermore, the Gr/CIP-PDMS multiphase interface exhibits a significant Maxwell–Wagner polarization effect under high-frequency electromagnetic excitation, causing charge to accumulate and relax at the interface, thereby further enhancing dielectric polarization losses. Therefore, the synergistic magnetic–dielectric loss effect of high-content Gr/CIP and the multilevel scattering paths of structural gradients synergistically enhance the absorption performance of broadband and efficient EMWs and are expected to provide an engineering-feasible solution for ultra-wideband electromagnetic protection and device integration.

### Broadband Absorption Properties of GCH Absorber

**Fig. 4:**
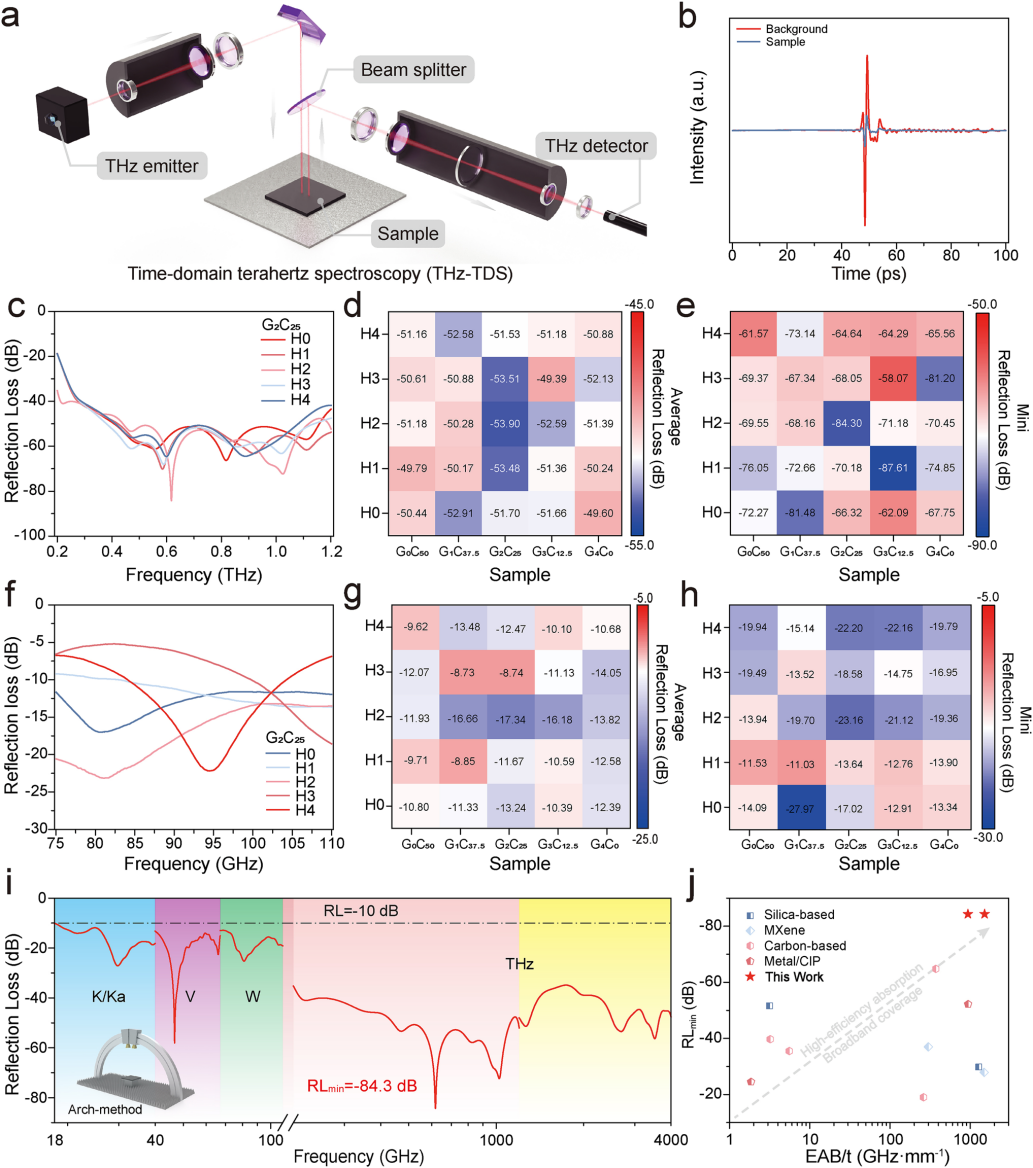
Broadband electromagnetic absorption performance of GCH absorber. **a** Schematic diagram of THz-TDS. **b** Schematic comparison of terahertz time-domain transmission pulses of air background and tested sample. **c** RL curves of H0-H4 structure of G_2_C_25_ in 0.2–1.2 THz. **d**–**e **Thermal maps of RL_ave_ and RL_min_ distribution for different formulations and structures. **f** RL curves of H0–H4 structure of G_2_C_25_ in 75–110 GHz. **g**–**h** Corresponding thermal maps of RL_ave_ and RL_min_ distribution. **i** RL combination curves of GCH-based absorbers in representative broadband ranges from GHz to THz bands. **j** Comparison of the RL_min_ and EAB/*t* with previously reported absorbers based on different material systems [56, 64–72]

To characterize the intrinsic electromagnetic response of GCH absorbers in the terahertz band, this study employed a time-domain terahertz spectroscopy (THz–TDS) system to test the samples [58, 59]. This system consists of a terahertz pulse source, optical path modulation components, and a detector acquires the time-domain transmission signal of the material, serving as an important tool for analyzing high-frequency electromagnetic properties (Fig. 4a). Figure 4b shows the time-domain pulse response of the test sample and air medium under terahertz excitation. Compared with the air reference signal, the electromagnetic properties of the material were calculated by observing the amplitude attenuation and time delay of the transmitted pulse. Based on this, the dielectric response of the GC series inks was tested (Fig. S9a–c). With the increase in the Gr mass ratio, the real part of the dielectric ε’ gradually increased, with the ε' of G_4_C_0_ inks exceeding 30, indicating that the introduction of Gr significantly enhanced the material’s polarization response and charge storage capability. Meanwhile, the values of the imaginary dielectric part ε" and the loss tangent tan*δ*_ε_ first increase and then decrease within the range of 0.2–1.2 THz. G_2_C_25_ exhibits the strongest loss capability, which is due to its combination of multiple loss mechanisms provided by Gr and CIP, including free carrier loss, interface polarization, and dipole relaxation. Dielectric relaxation in G_2_C_25_ was further assessed via Cole–Cole analysis in the 0.2–1.2 THz range (Fig. S9d). The resulting nonideal Cole–Cole arc and tailing feature suggest coexisting polarization relaxation and conductive loss. Specifically, defects and residual polar groups on Gr contribute to dipole polarization, while multiphase heterointerfaces induce Maxwell–Wagner–Sillars polarization through local charge accumulation. Concurrently, interconnected Gr sheets facilitate carrier transport, enhancing conductive loss [60]. Thus, the robust dielectric loss of G_2_C_25_ stems from the synergy between dipole/interfacial polarization and conduction. Furthermore, the absorption performance of structured GCH absorbers in the terahertz band was investigated. Using G_2_C_25_ ink as an example, the RL curves of five honeycomb gradient structures H0–H4 within the range of 0.2–1.2 THz were analyzed (Fig. 4c). Clearly, the RL of all structures in the terahertz band is below − 20 dB. This indicates that structural gradients can significantly improve energy loss efficiency in the terahertz range by extending the propagation path, enhancing scattering, and smoothing impedance transition. Among them, the H2 gradient structure exhibits an RL_ave_ below − 35.2 dB and an RL_min_ reaching − 84.30 dB in the terahertz band. To further elucidate this structural enhancement effect, the dielectric parameters and equivalent impedance matching |*Z*_in_/*Z*_0_| behavior of H0–H4 were analyzed (Fig. S10). Clearly, compared to the G_2_C_25_ film, the real part ε', imaginary part ε", and dielectric loss tangent of H0–H4 are significantly reduced. Simultaneously, the equivalent impedance matching |*Z*_in_/*Z*_0_| is optimized to near 1, exhibiting near-perfect matching. Specifically, the equivalent impedance of H0 exhibits a larger amplitude in the high-frequency range of 0.8–1.2 THz, while H4 shows a larger oscillation amplitude in the low-frequency range of 0.2–0.6 THz. H2 maintains optimal matching across the entire bandwidth, consistent with its extremely low RL. Furthermore, Fig. S11a–d investigates the RL variation of GCH absorber in the 0.2–1.2 THz under different Gr contents. The data show that the position of the reflection loss peak can be modulated by changing the Gr content and structural gradient, and the absorption intensity remains basically below − 30 dB. An appropriate Gr content can enhance the dielectric loss capability of the material at higher frequencies, exhibiting stronger electromagnetic wave attenuation capability [61]. Further thermographic analysis of RL_ave_ and RL_min_ of GCH was performed (Fig. 4d, e) to reveal the synergistic effect of ink composition and gradient honeycomb combination. The optimal performance in the terahertz range is concentrated in the optimal region centered on *G*_2_*C*_25_–*G*_3_*C*_12.5_ × *H*2–*H*4, with RL_ave_ exceeding − 53.90 dB and RL_min_ below − 84.30 dB. In contrast, the G_4_C_0_ formulation, due to excessive conductivity, leads to impedance mismatch, resulting in a decrease in absorption performance.

To investigate the absorption behavior of the material in a wider frequency band, the RL curves of the H0–H4 structures in the *W* band were tested using *G*_2_*C*_25_ ink as an example (Fig. 4f). Unlike the mechanism dominated by multilevel scattering in the terahertz band, the structure size is comparable to the wavelength in the millimeter band, which leads to enhanced local resonance of Fabry–Pérot, manifested as the appearance of resonance absorption peaks [62]. The resonance absorption peak of the small-sized H0 honeycomb structure is located at 80.77 GHz (− 17.02 dB), while the resonance absorption peak of the large-sized H4 honeycomb structure is located at 94.42 GHz (RL_min_ reaches − 22.20 dB). In comparison, the RL_min_ of H2 can reach − 23.16 dB at 82.29 GHz, and the overall absorption performance is improved. Furthermore, Fig. 4g–h shows the RL_ave_ and RL_min_ of the GCH absorber in the *W* band for different ink compositions and gradient honeycomb combinations. Among them, G_2_C_25_H2 has the lowest RL_ave_, reaching − 17.34 dB, while G_1_C_37.5_H0 has an RL_min_ of − 27.97 dB. Figure S12 shows the RL curves for other formulations with different gradient honeycomb combinations in the W band. Overall, the high absorption efficiency of the GCH absorber depends on the combined effect of material electromagnetic loss and structural gradient impedance optimization. Furthermore, to evaluate the electromagnetic stability of the GCH under complex operating conditions, its response characteristics to changes in incident angle, structural thickness, and polarization mode were analyzed (Fig. S13). Clearly, the RL curve shows a limited increase with increasing incident angle, while still maintaining continuous broadband effective absorption [63]. Simultaneously, as the number of printing layers increases from 1 to 5, the absorption peak gradually shifts to lower frequencies, and the absorption intensity significantly increases. Moreover, the RL curves remain highly consistent in both TM and TE modes.

To clarify the intrinsic attenuation mechanism of the G_2_C_25_ sample in the low-frequency range, its electromagnetic parameters were further measured across the 18–40 GHz range (Fig. S14). The frequency-dependent ε' and ε" exhibit dispersive dielectric characteristics (Fig. S14a), while the nonideal Cole–Cole arc indicates coexisting dielectric relaxation and conductive loss (Fig. S14b). Meanwhile, the *μ*' and *μ*" spectra, together with the high magnetic loss tangent, confirm that CIP-related magnetic loss remains an important attenuation pathway (Fig. S14c, d). Therefore, the G_2_C_25_ sample provides synergistic magnetic–dielectric dissipation driven by interfacial/dipolar polarization, Gr-network conductive loss, and CIP-related magnetic loss. To improve impedance matching and absorption performance in the low-frequency range, a mesh matching layer (ML) based on G_2_C_25_ ink was introduced, taking advantage of the designability of the DIW process. Fig. S15a, b shows the porous ML, comprising periodic mesh units (*r* = 0.4 mm, *W* = 0.4–2.0 mm). This ML functions as an impedance transition layer that reduces surface reflection and facilitates EMWs entry into the high-loss G_2_C_25_H2 absorber. Tuning *W* optimizes the low-frequency RL; notably, *W* = 1.2 mm enables effective absorption across 18–40 GHz (Fig. S15c). The test results in Fig. S16 show that the ML significantly improves the insufficient low-frequency absorption of the original GCH, achieving continuous and effective absorption from 18 to 40 GHz. Further testing yielded the RL curves of G_2_C_25_H2 in the D/G bands (110–170 GHz, 170–220 GHz) and 1.2–4.0 THz range (Fig. S17). The D/G-band results satisfied the effective absorption criterion (Fig. S17a, b), and even at higher THz frequencies, the RL of G_2_C_25_H2 remained below − 35 dB (Fig. S17c). Broadband RL measurements showed that the G_2_C_25_H2-based GCH-ML configuration provides effective absorption over 18–67 GHz, whereas the corresponding standalone 2.6-mm-thick GCH absorber without ML is sufficient to cover 67–4000 GHz (Figs. 4i and S17). Through the combined effects of material loss optimization, structural gradient design, and low-frequency matching layer regulation, the 3D-printed absorber achieved continuous and effective absorption across 18 GHz–4 THz with a maximum thickness of only 4.25 mm (Figs. 4i and S17). Its RL_min_ reached − 84.30 dB, and the RL_ave_ was − 31.4 dB. Considering that practical device-level integration requires reliable environmental durability, broadband RL measurements were conducted after long-term air exposure, hygrothermal aging, and mechanical handling tests (Fig. S18). After exposure to air for six months followed by 168 h of hygrothermal aging at 85 °C/85% relative humidity (RH), effective absorption was largely maintained over the measured broadband ranges. Similarly, after 200 bending-release cycles at a radius (R) of 8.5 mm, the RL response remained largely stable, demonstrating good durability against environmental aging and mechanical handling. To benchmark diverse absorber systems [56, 64–72], the effective absorption bandwidth per unit thickness (EAB/t) was adopted. The GCH absorber (Fig. 4j and Table S3) exhibits a favorable balance between EAB/t and RL_min_, highlighting its integrated broadband attenuation capability and 3D-printable structural merits.

### EMC Application Verification of GCH Absorber

**Fig. 5:**
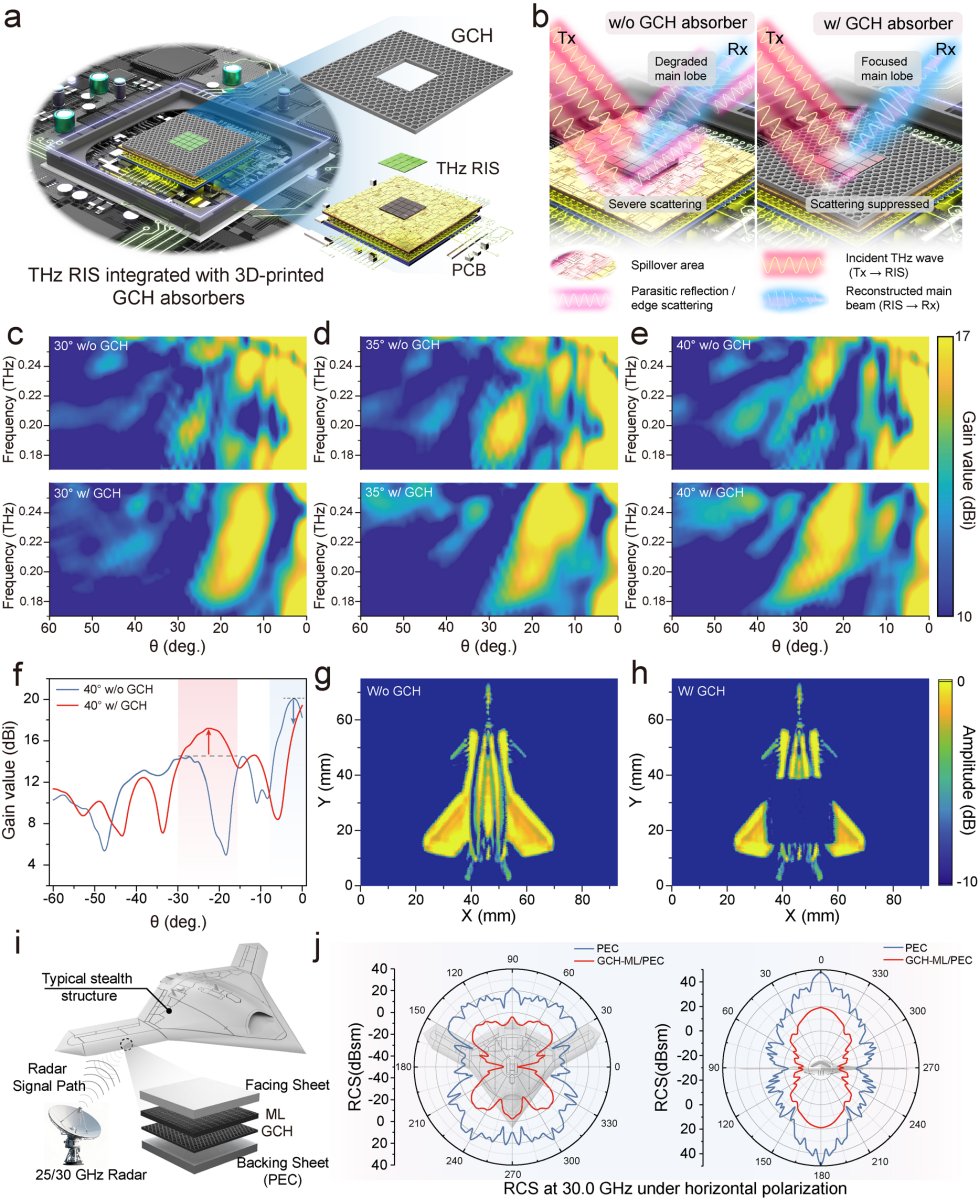
Application verification of the 3D-printed GCH absorber. **a** Schematic diagram of the integration of GCH with THz RIS. **b** GCH absorber suppresses parasitic reflections and purifies the local electromagnetic environment of the RIS. **c**–**e** Comparison of frequency–angle gain distribution with and without GCH at different scanning angles (30°, 35°, 40°). **f** Comparison of gain angle curves under 40° scanning condition at 220 GHz. **g**–**h** Comparison of 120 GHz radar imaging results with and without GCH coverage. **i** Schematic diagram of GCH-ML integrated as a functional layer in a typical stealth structure. **j** Comparison of RCS polar coordinates between pure PEC and GCH-ML/PEC structure under 30 GHz horizontal polarization condition

Terahertz RIS utilize periodic reflective arrays to perform phase encoding and beam reconstruction of incident EMWs, making them critical components in future high-frequency wireless communication and beamforming systems [73, 74]. However, the metal frame, feed structure, and finite edges inevitably induce significant parasitic reflections and edge scattering, thereby degrading the local electromagnetic environment and limiting main lobe purity and side lobe suppression [75, 76]. Leveraging DIW 3D printing conformal technology [25, 36], the GCH absorbing structure can be constructed precisely in situ around the RIS array. This integration method avoids the gaps and assembly errors inherent in traditional bonding techniques (Figs. 5a and S19a). By absorbing these stray reflections, the system achieves reduced background scattering, enhanced side lobe suppression, and improved main lobe clarity, thereby optimizing both the local radiation environment and beam reconstruction quality (Figs. 5b and S19b). Beam scanning test results at different encoding angles (Fig. 5c–e) confirm the significant enhancement of RIS radiation characteristics provided by the 3D-printed absorber. Without absorption integration, pronounced electromagnetic interference appears in the 2D beam patterns as a bright background band and scattered patches. Specifically, a persistent, bright “specular reflection” region, spanning 7°–8° near 0°, makes distinguishing the main lobe from the side lobes challenging. In addition, spurious radiation leads to significant main lobe beam splitting, which in turn results in a severe reduction in scanning bandwidth. Upon integrating the structured 3D-printed absorbers, stray reflections are substantially suppressed, background brightness is drastically reduced, and main lobe concentration sharply improves. The main lobe becomes significantly more concentrated and directionally stable across all coding angles. The average main lobe gain increased by ~ 3.3 dBi, reaching approximately 16 dBi (Figs. 5f and S20a, b). Simultaneously, average side lobe gain decreased by 0.2–1.6 dBi, resulting in the main lobe-side lobe level difference increasing to 1.9–3.3 dB (Fig. S20c). Moreover, the specular reflection amplitude was reduced by 0.4–1.1 dBi (Fig. S20a, b) and its beamwidth was significantly narrowed to 3° (a reduction exceeding ~ 58%) (Fig. S20d). This confirms the absorbing structure’s excellent suppression capabilities against oblique incidence (− 15°) reflections, effectively purifying the RIS array’s electromagnetic environment. Continuous main beam illumination drives primary RIS thermal loading via internal ohmic and dielectric dissipation. The peripheral GCH absorber, capturing only residual stray and edge-scattered waves, contributes marginally to self-heating. For reflective architectures, surface-level heat exchange is preferable to bulky heat sinks to avoid obstructing the incident/reflected wavefronts. Accordingly, an equivalent surface heat transfer experiment was designed (Fig. S21a). The GC sample shows faster heating and cooling responses than PDMS (Fig. S21b), owing to its much higher thermal conductivity (~ 2.5 vs. ~ 0.27 W m^−1^ K^−1^, Fig. S21c). Together with the enlarged exposed area of the hollow honeycomb structure, the GCH absorber can help mitigate local thermal accumulation during RIS integration. Collectively, these results demonstrate that the conformally integrated GCH absorber effectively suppresses RIS edge scattering and significantly optimizes beam quality and pointing performance, offering a feasible approach for device-level electromagnetic compatibility (EMC) optimization of THz RIS.

Furthermore, Fig. 5g–h shows the imaging results of the aircraft model without and with GCH absorber under 120 GHz radar imaging conditions (the test scenario is shown in Fig. S22). Without the GCH absorber, the aircraft surface exhibits a strong bright scattering area, especially concentrated at the fuselage edge, corner, and structural change points. These areas are prone to specular reflection and diffraction effects, resulting in a significant increase in RCS intensity [77]. After introducing the GCH absorber, the high intensity scattering area is effectively weakened, indicating that the incident 120 GHz electromagnetic wave is absorbed and dissipated. This result intuitively proves the effective control capability of GCH on the scattering characteristics of complex target surfaces in the sub-terahertz band. Furthermore, Fig. 5i shows the application of GCH-ML as a functional layer integrated into a typical stealth structure. Under 30 GHz horizontal polarization conditions, the GCH-ML/PEC composite structure exhibits significantly lower scattering intensity than the pure PEC structure in all azimuth angle ranges (Fig. 5j), and the RCS is reduced by up to 21.4 dBsm. This RCS simulation evaluates the target-level electromagnetic scattering suppression potential of the GCH absorber and demonstrates its feasibility for localized or modular electromagnetic functional-layer integration on conformal surfaces. In summary, GCH-ML absorbers with wideband loss characteristics and excellent structural designability have demonstrated significant engineering potential in applications such as THz communication, radar imaging, and electromagnetic scattering suppression.

## Conclusions

This study addresses the challenges of processability and geometric controllability in the structured manufacturing and integrated deployment of high-load magneto-dielectric absorbing materials under device-level electromagnetic compatibility (EMC) requirements. A direct ink writing (DIW) design and manufacturing strategy based on rheological engineering is proposed and validated. By introducing graphene (Gr) to reconstruct the carbonyl iron powder (CIP) particle network, a multi-dimensional Gr/CIP synergistic load-bearing network is formed. This achieves synergistic enhancement of high-load ink shear-thinning behavior, yield characteristics, and structural recovery capability without relying on chemical crosslinking or low-load formulations. Based on the yield model and deposition stability criterion, a quantitative correlation between ink composition, rheological response, and printability is established, clarifying the predictive ability of the rheological window on extrusion filamentation, 2D geometric fidelity, and 3D temporal stability. The 3D-printed GCH absorber achieves ultra-wideband electromagnetic absorption (RL ≤ − 10 dB) covering 18 GHz–4 THz, and the RL_min_ reaches − 84.30 dB in the terahertz band with a thickness of only 2.6 mm. Device-level integration experiments demonstrate that the GCH absorber can simultaneously improve the main lobe gain (~ 3 dBi) and sidelobe suppression (1.9–3.3 dB) of terahertz RIS, and reduce the specular reflection beamwidth by ~ 58%. It also exhibits stable scattering suppression capabilities under millimeter-wave imaging/radar conditions, demonstrating its good system compatibility and engineering applicability. This research shows that material structure co-design based on rheological engineering provides a predictable and scalable path for the design and fabrication of high-load functional composite materials and is expected to drive the transformation of absorbing materials from performance-oriented research to device-level integrated applications.

## Supplementary Information

Below is the link to the electronic supplementary material.

Supplementary file1 (DOCX 30723 KB)
