# Supplementary material for: Rheologically Engineered 3D-Printed Highly Loaded Magneto-Dielectric Absorbers for Device-Level Electromagnetic Compatibility
Source: Nanomicro Lett. 2026 Jul 30;19:13. doi: 10.1007/s40820-026-02312-7 (PMC13424068; doi:10.1007/s40820-026-02312-7)
Supplement: Supplementary file 1 — Supplementary file1 (DOCX 30723 KB) [file 40820_2026_2312_MOESM1_ESM.docx]

Supporting Information for

**Rheologically Engineered 3D-Printed Highly Loaded Magneto-Dielectric Absorbers for Device-Level Electromagnetic Compatibility**

Yuheng Jiang^1, †^, Zihao Chen^1, †^, Xiao Sun^1, 2,^ *, Haotian Li^1^, Jinlong Xie^1^, Yueting Li^1^, Xiaolei Nie^1^, Feng Lan^1, 3,^ *, Yaxin Zhang^1, 3^ *, Qiye Wen^1, 2, 3^ *

^1^ School of Electronic Science and Engineering, University of Electronic Science and Technology of China, Chengdu 611731, China

^2^ Shenzhen Institute for Advanced Study, University of Electronic Science and Technology of China, Shenzhen 518110, China

^3^ Engineering Center of Integrated Optoelectronic & Radio Meta-chips, Chengdu, 611731, China

^†^ Yuheng Jiang and Zihao Chen contributed equally to this work.

*Corresponding author. E-mail: [qywen@uestc.edu.cn](mailto:qywen@uestc.edu.cn) (Qiye Wen), [langfeng@uestc.edu.cn](mailto:langfeng@uestc.edu.cn) (Feng Lan), [xiaosun@uestc.edu.cn](mailto:xiaosun@uestc.edu.cn) (Xiao Sun), [zhangyaxin@uestc.edu.cn](mailto:zhangyaxin@uestc.edu.cn) (Yaxin Zhang)

**The content of supplementary material:**

Total number of pages: 13

Total number of figures: 22

Total number of tables: 3

**Supplementary Figures and Tables**


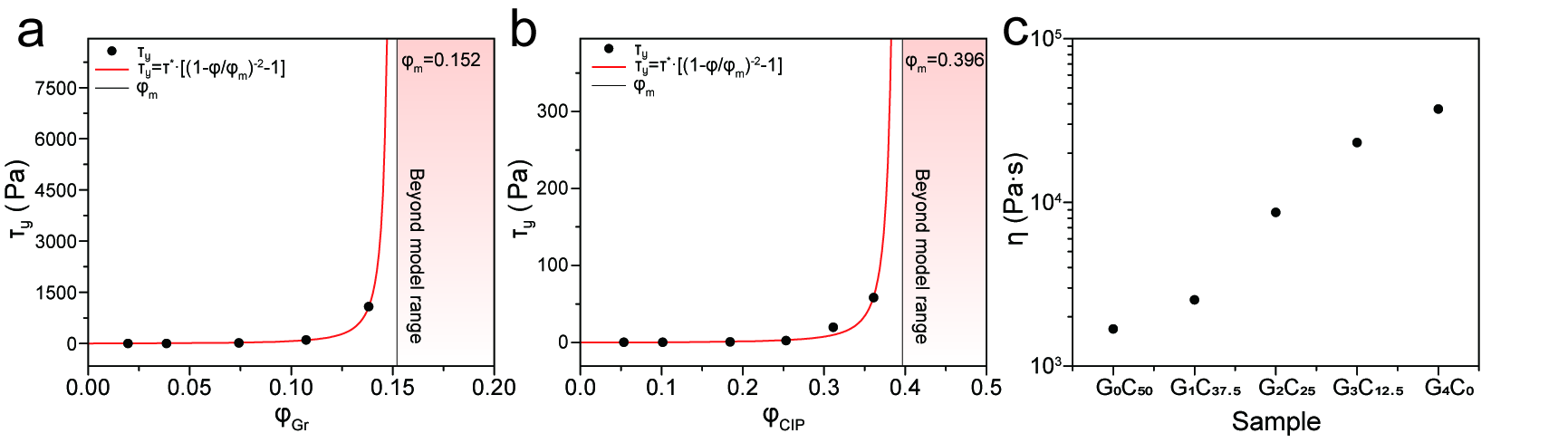


**Figure S1.** (a) The relationship between τ_y_ and φ_Gr_ in Gr/PDMS ink, fitted by the Heymann model. (b) The relationship between τ_y_ and φ_CIP_ in CIP/PDMS ink, fitted by the Heymann model. (c) η of GC ink with endpoints from G_0_C_50_ to G_4_C_0_ at low shear rates (0.01 s^-1^).


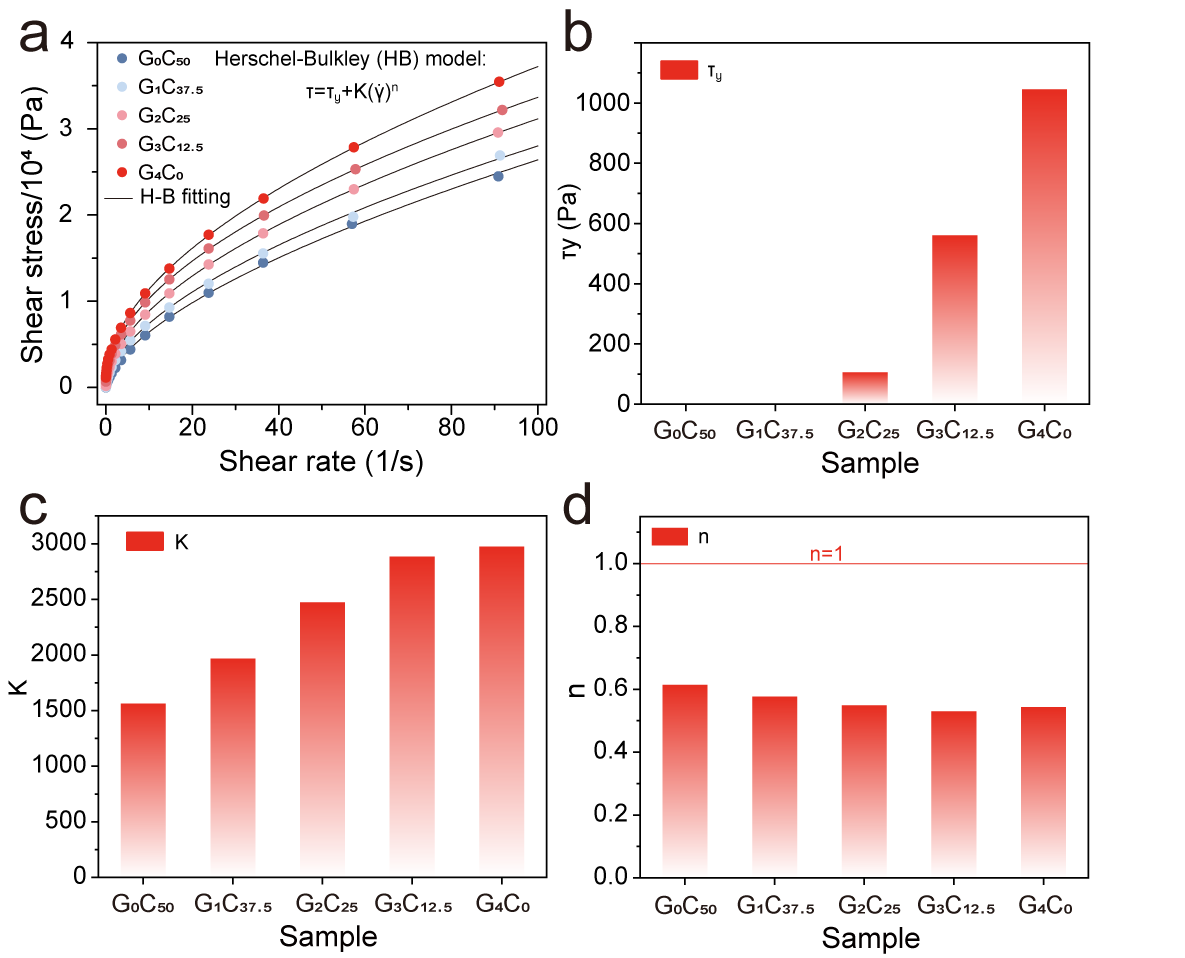


**Figure S2.** (a) Shear stress-shear rate curves and Herschel-Bulkley model fitting results. (b) τ_y_ obtained from the H-B model fitting as a function of GC ink composition. (c) Corresponding consistency coefficient K. (d) Rheological index n.


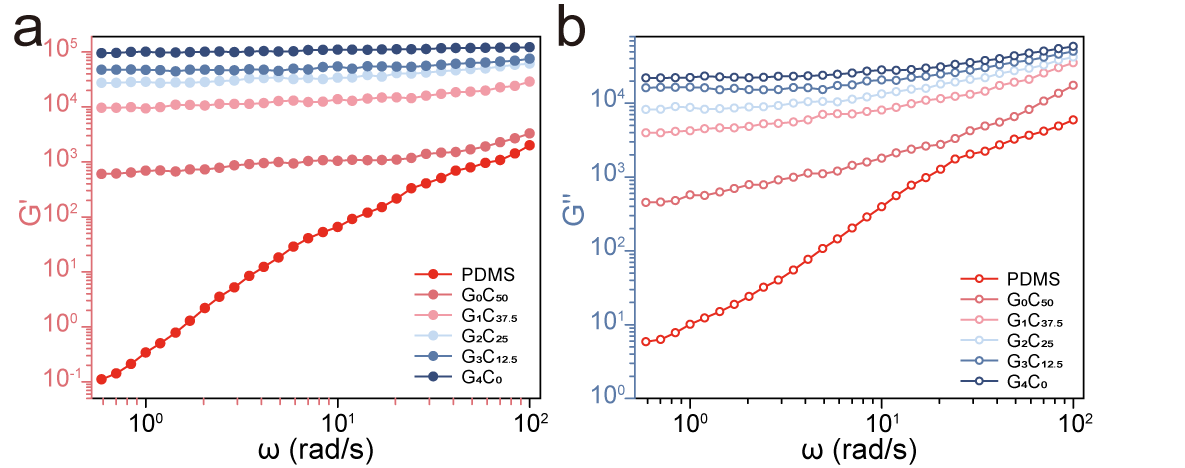


**Figure S3.** Rheological results from frequency sweeps of PDMS and GC inks: (a) storage modulus G' and (b) loss modulus G'' as functions of angular frequency (ω).

Compared with PDMS and G_0_C_50_, Gr-containing GC inks exhibit higher G' and G'' values with weaker frequency dependence, indicating the formation of a more stable physical network and a slower relaxation process.


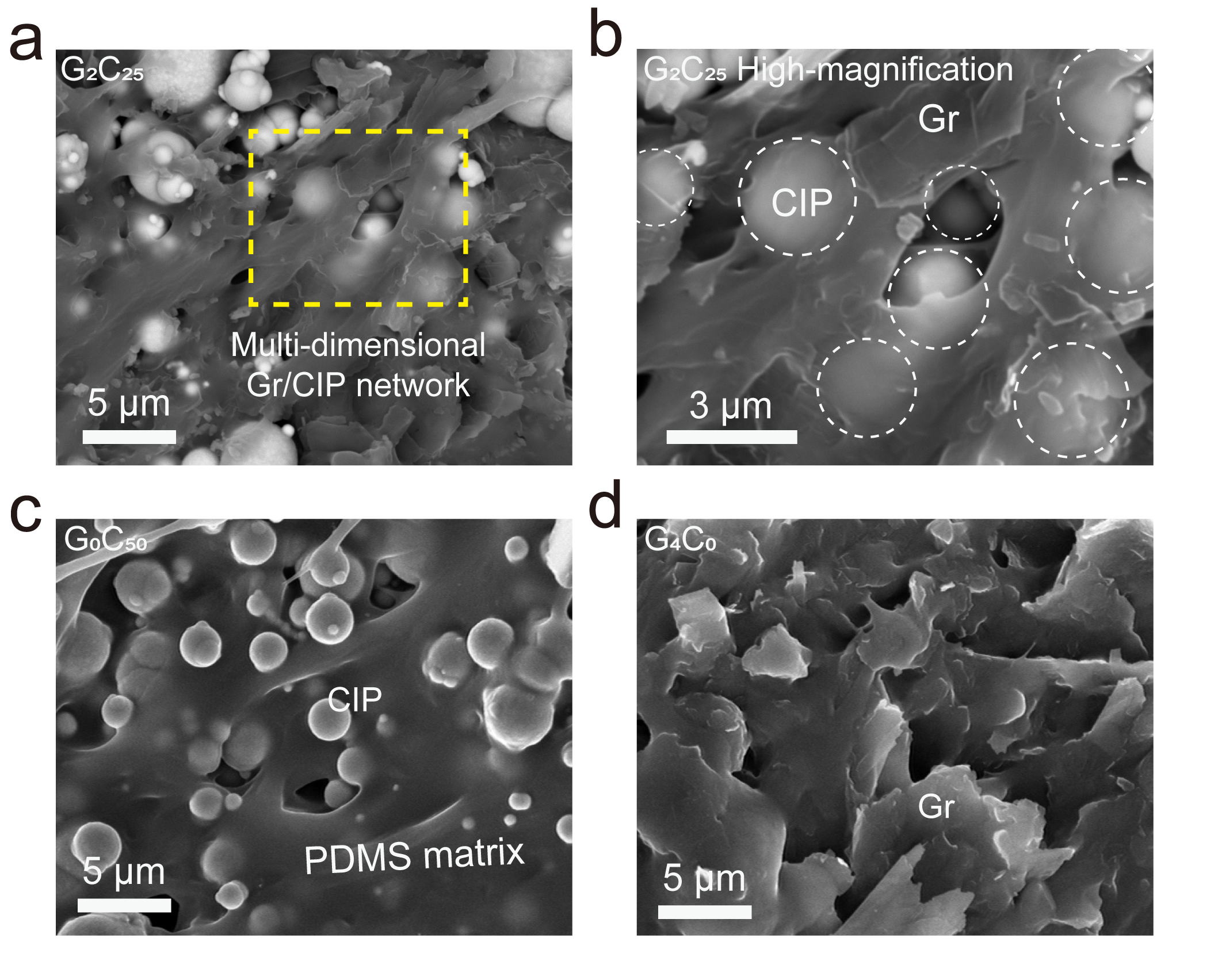


**Figure S4.** Cross-sectional SEM images of representative GC samples: (a), (b) G_2_C_25_, (c) G_0_C_50_, and (d) G_4_C_0_.

G_0_C_50_ is dominated by spherical CIP particles dispersed in the PDMS matrix, while G_4_C_0_ shows a sheet-like Gr network. In G_2_C_25_, spherical CIP particles are spatially embedded among interconnected Gr sheets, confirming the formation of a representative multi-dimensional Gr/CIP network.


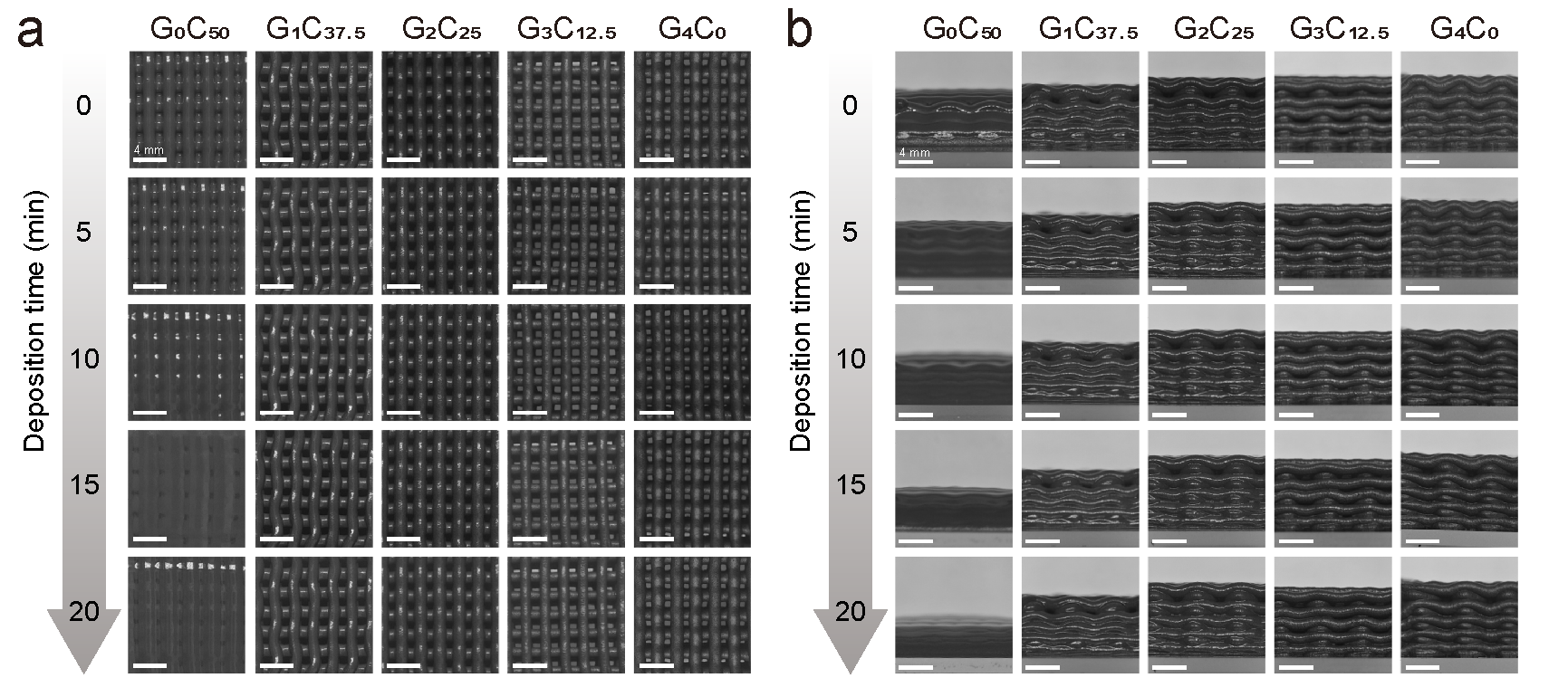


**Figure S5.** Top (a) and side (b) optical photographs of the corresponding GC series samples at different deposition times.


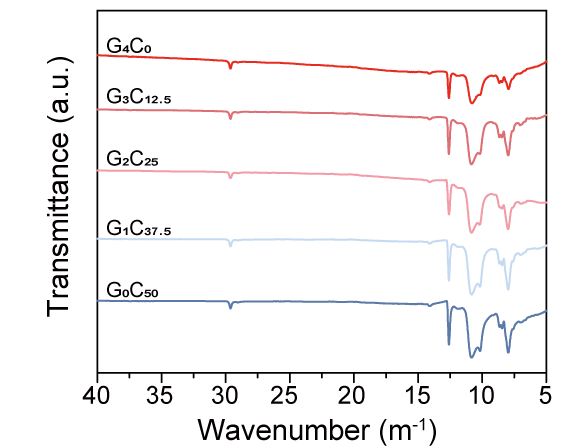


**Figure S6.** FTIR spectra of samples with different Gr/CIP ratios.


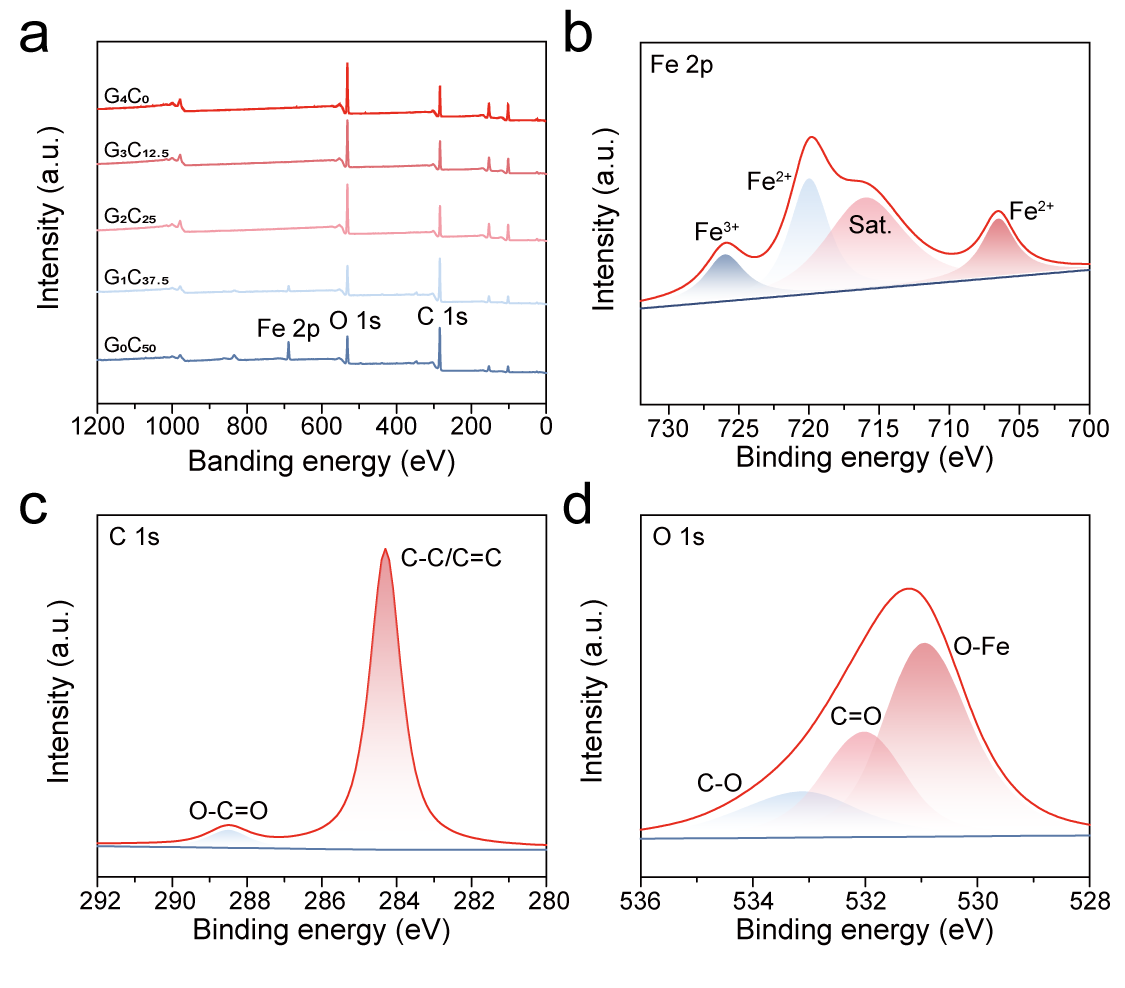


**Figure S7.** (a) XPS full spectrum of samples with different Gr/CIP ratios. (b)–(d) High-resolution XPS results for Fe 2p, C 1s and O 1s, respectively.


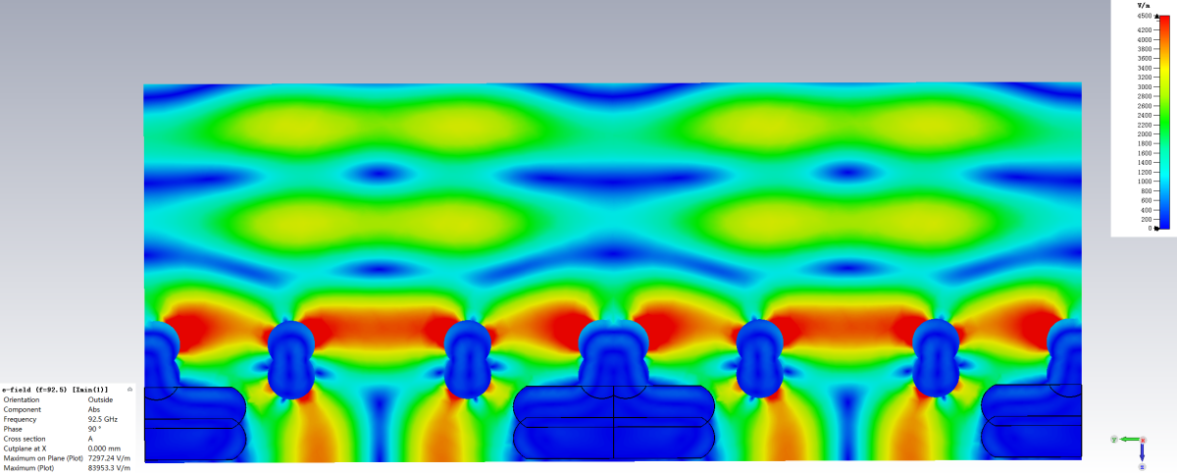


**Figure S8.** Electric field distribution of 3D-printed GCH absorber under 90 GHz.


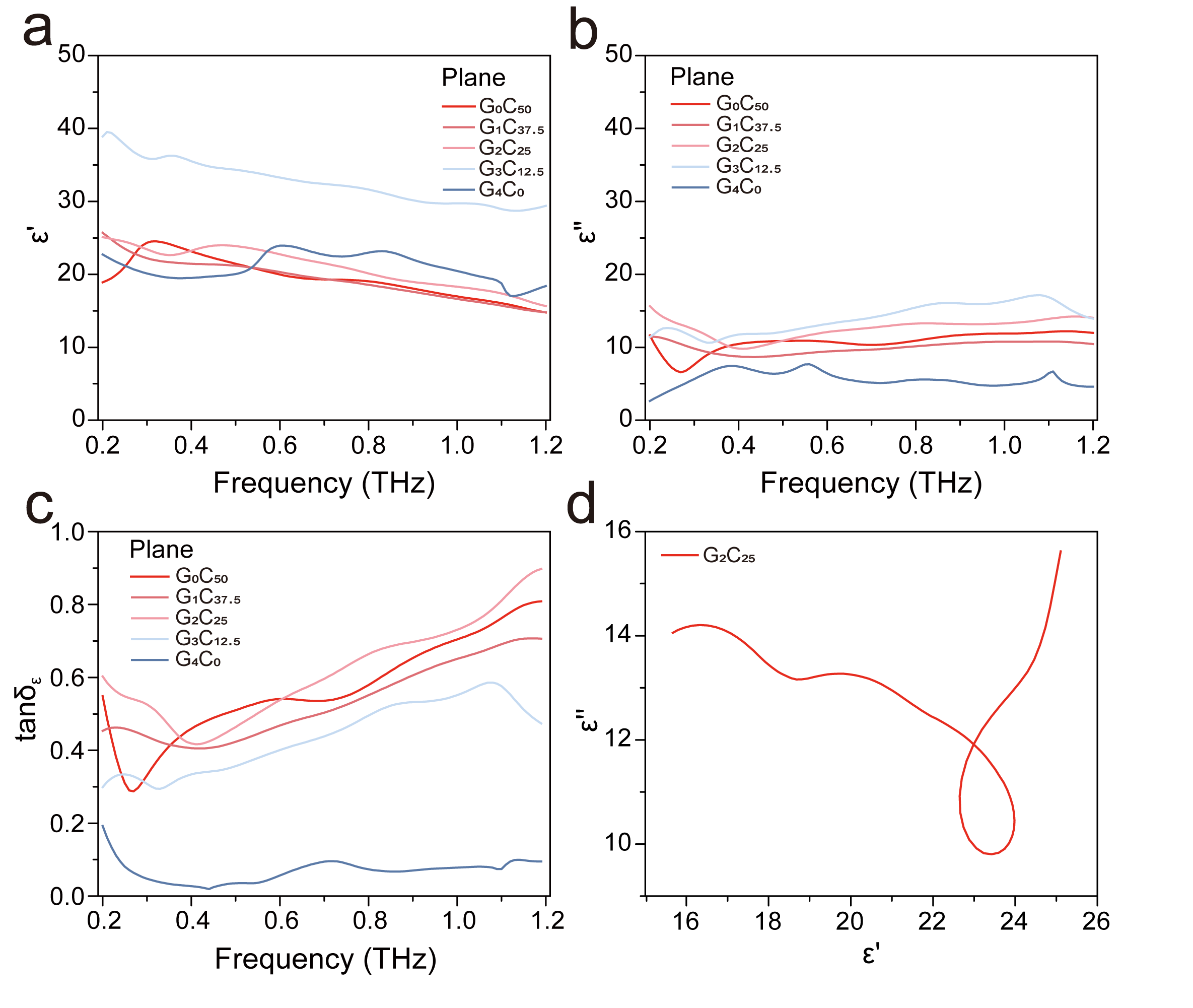


**Figure S9.** (a)–(c) Relationship between the real part ε', imaginary part ε", and loss tangent tan δ_ε_ of planar samples with different GC formulations in the 0.2–1.2 THz band. (d) Cole-Cole plot of the representative G_2_C_25_ sample in the 0.2–1.2 THz band.


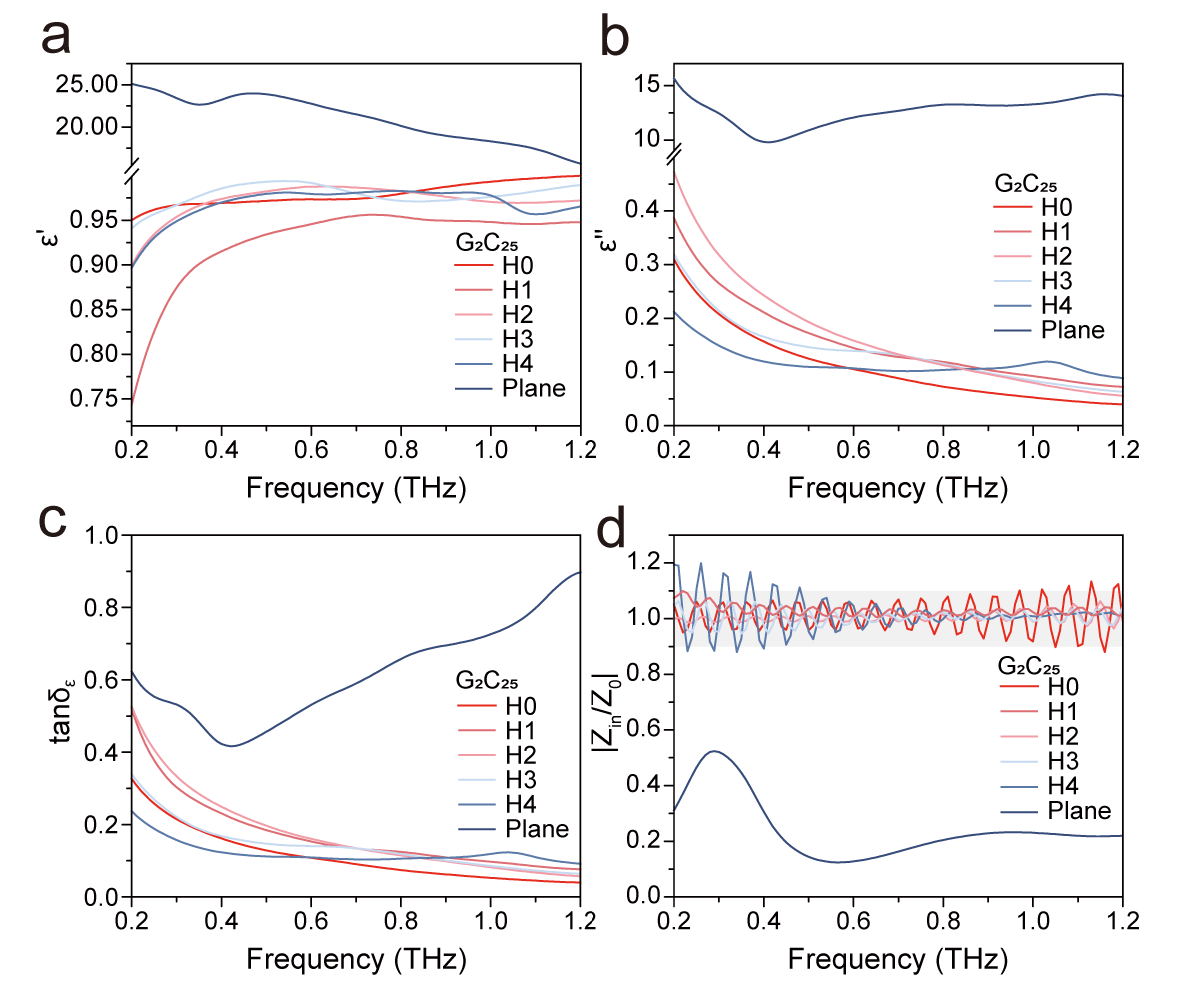


**Figure S10.** (a)-(c) Equivalent dielectric parameters ε', ε" and tan δ_ε_ of G_2_C_25_ formulation under different cellular gradient structures (H0–H4) and planar structures. (d) Normalized input impedance |Z_in_/Z_0_| of the corresponding structure as a function of frequency.


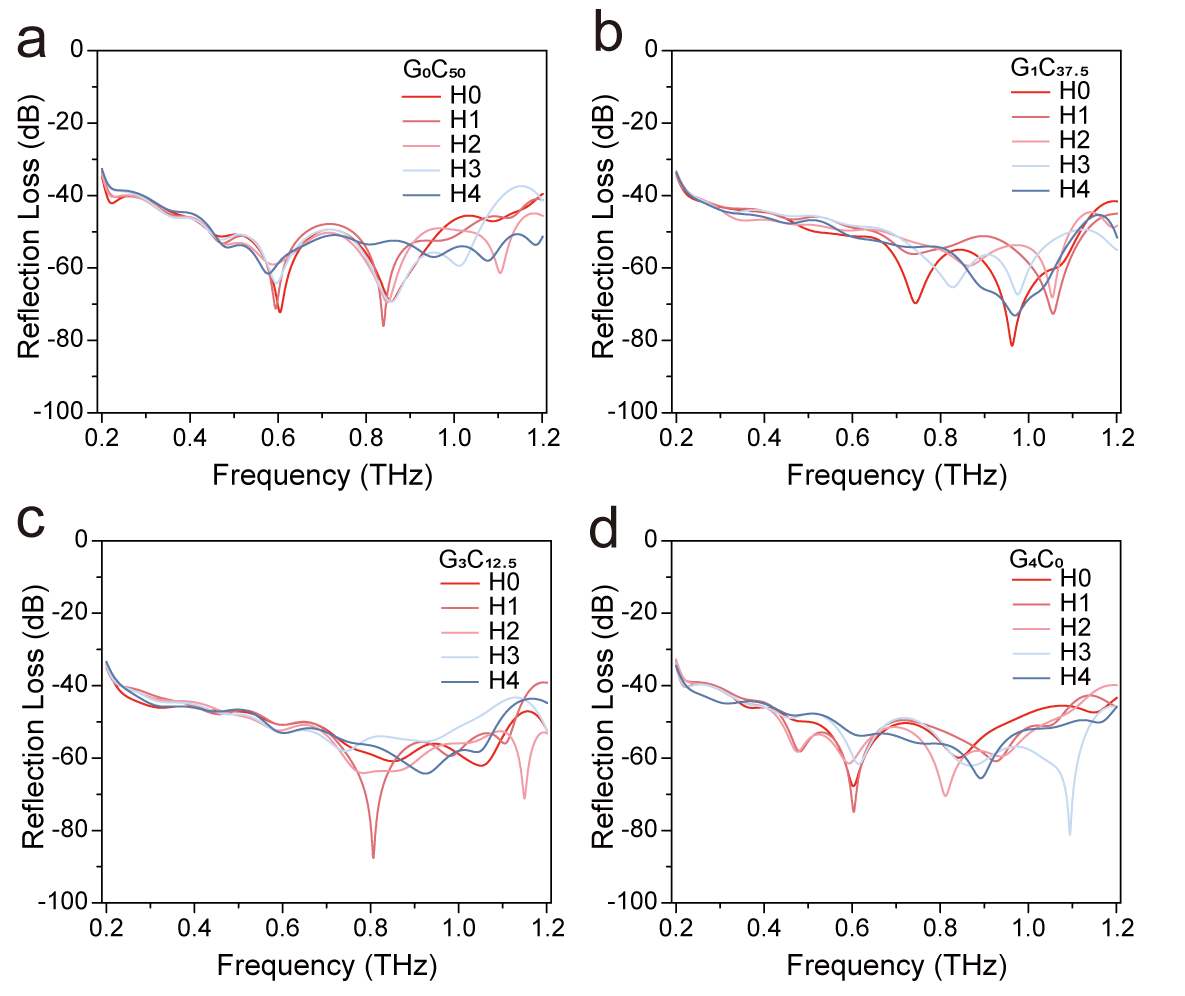


**Figure S11.** (a)–(d) RL curves of the honeycomb gradient structures (H0–H4) with different formulations (excluding G_2_C_25_) in the 0.2–1.2 THz.


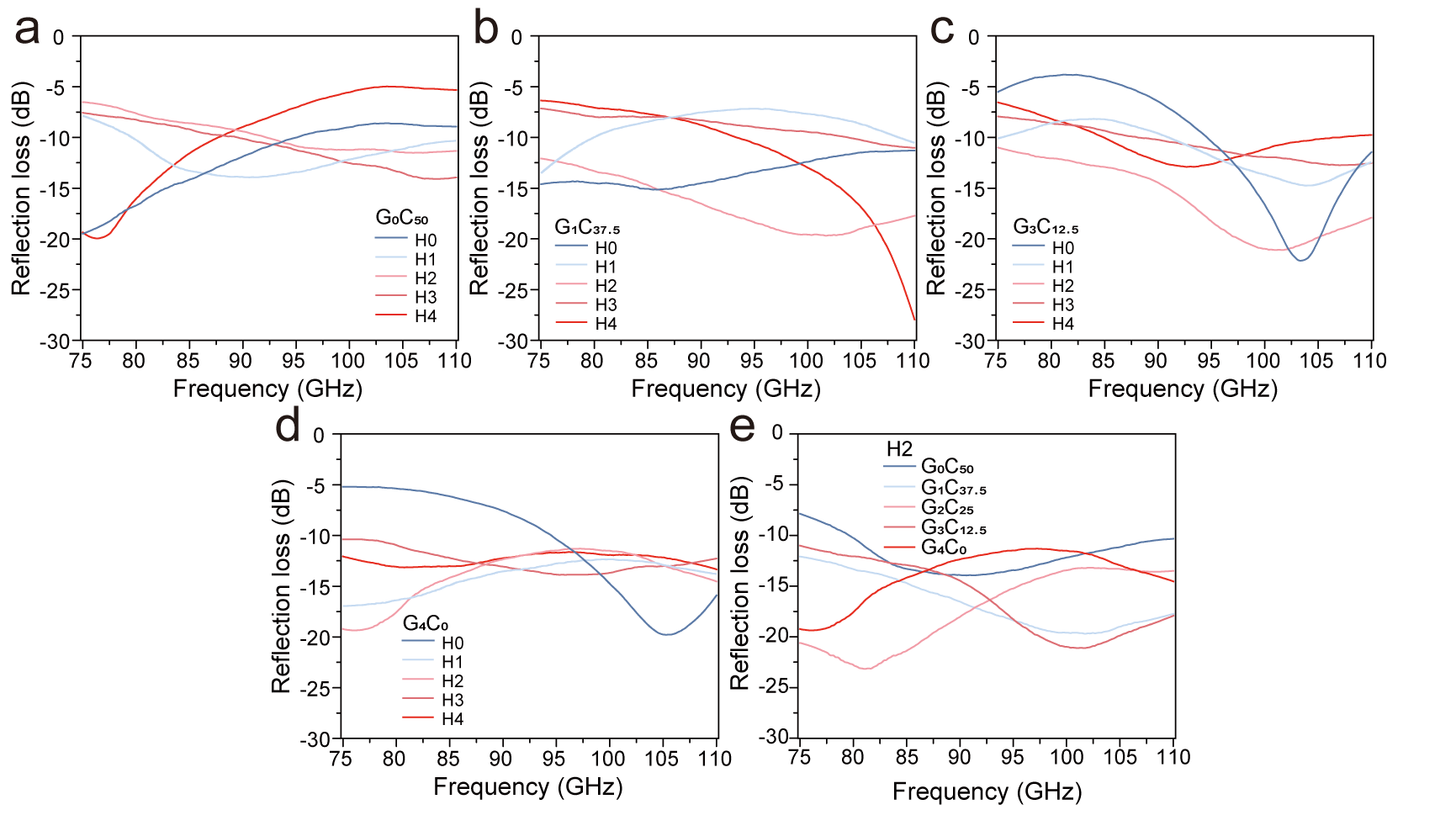


**Figure S12.** (a)–(d) RL curves for gradient structures (H0–H4) with different formulations (excluding G_2_C_25_) in the 75–110 GHz. (e) Comparison of absorption performance of different formulations in the 75–110 GHz under a fixed H2 structure.


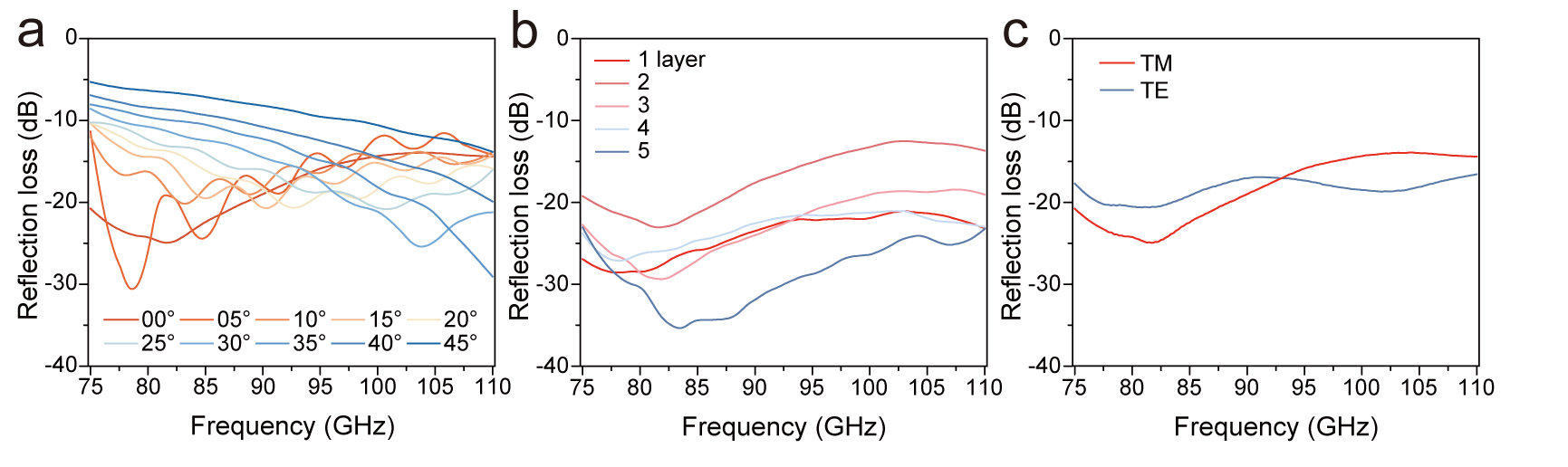


**Figure S13.** (a) RL response under different incident angles (0°–45°). (b) RL variation under different number of printed layers. (c) Comparison of RL in TE and TM polarization modes.


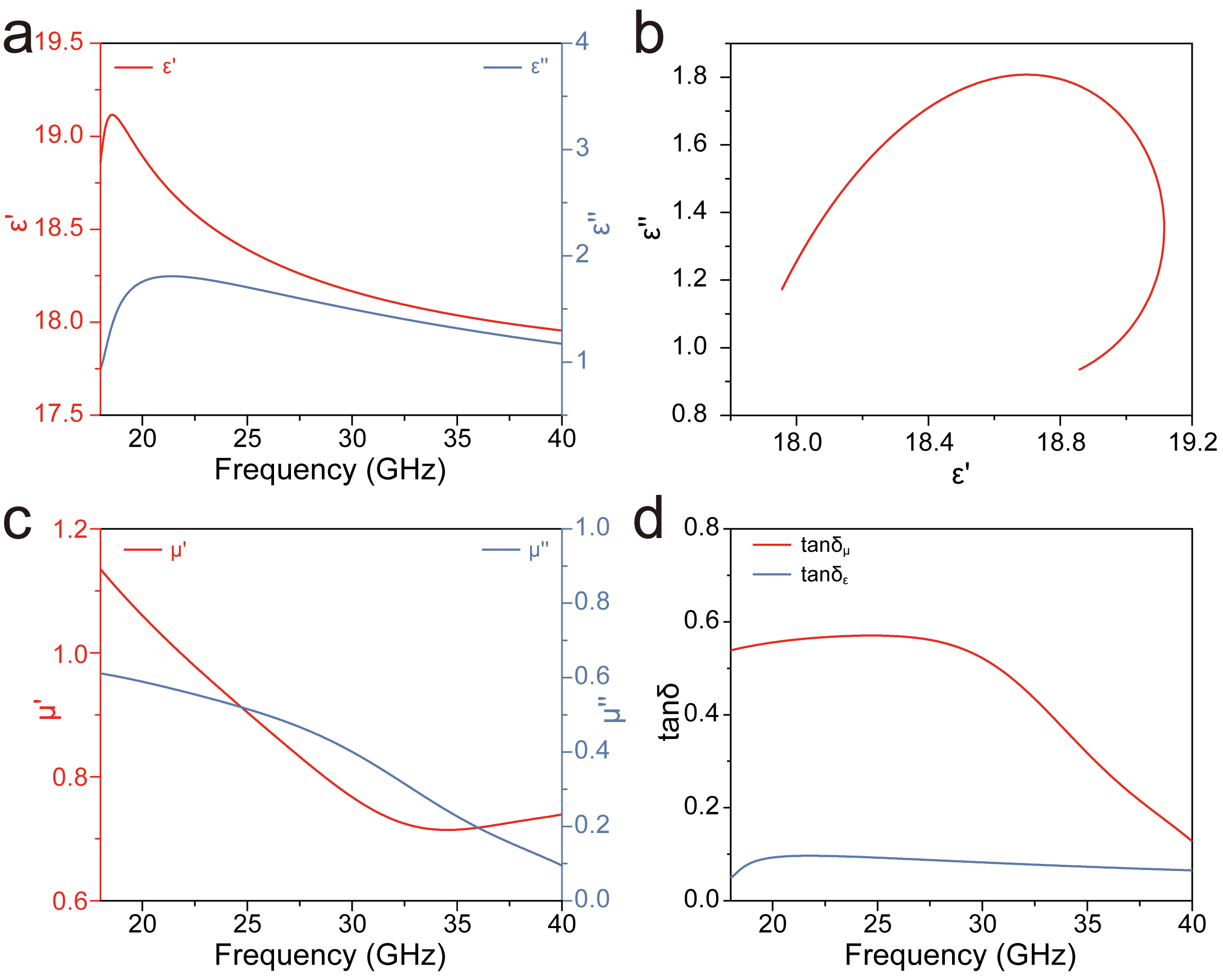


**Figure S14.** Electromagnetic parameters and Cole-Cole analysis of G_2_C_25_ in the 18–40 GHz frequency band: (a) ε' and ε'', (b) Cole-Cole plot, (c) μ' and μ'', (d) dielectric and magnetic loss tangents.


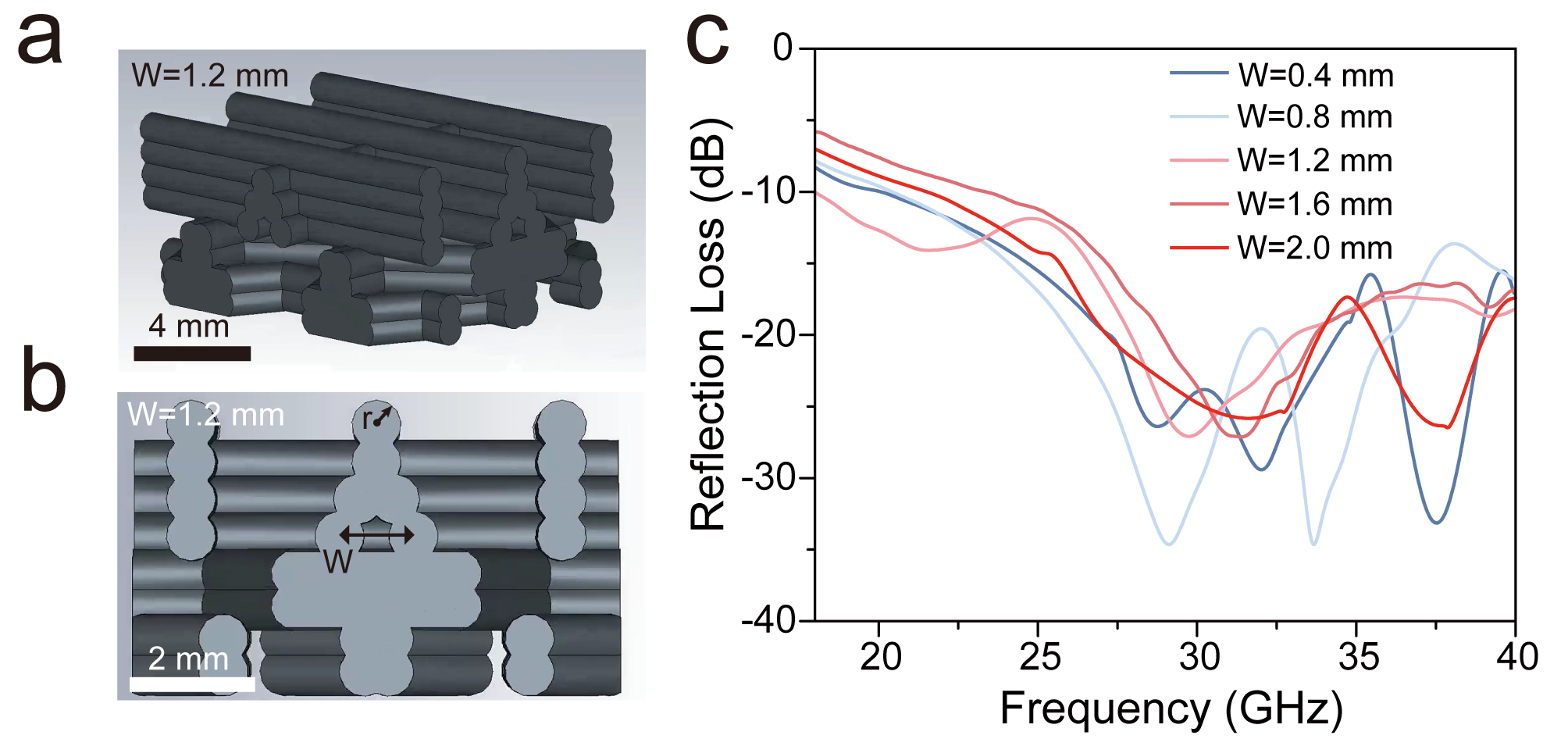


**Figure S15.** (a) Schematic diagram of the three-dimensional structure of the mesh matching layer. (b) Schematic diagram of the side view structure. (c) RL curves in the range of 18–40 GHz with different aperture widths (W).


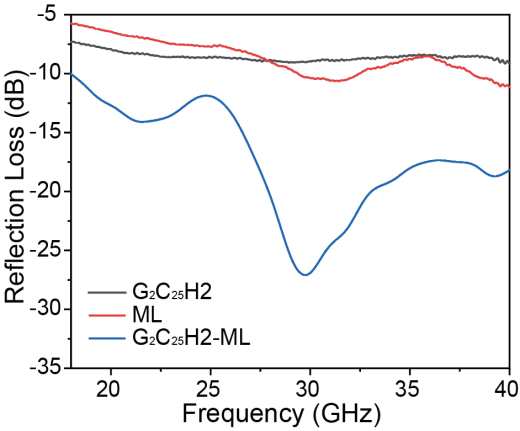


**Figure S16.** Comparison curves of RL for the G_2_C_25_H2 structure, matching layer (ML), and G_2_C_25_H2-ML composite structure in the 18–40 GHz band.


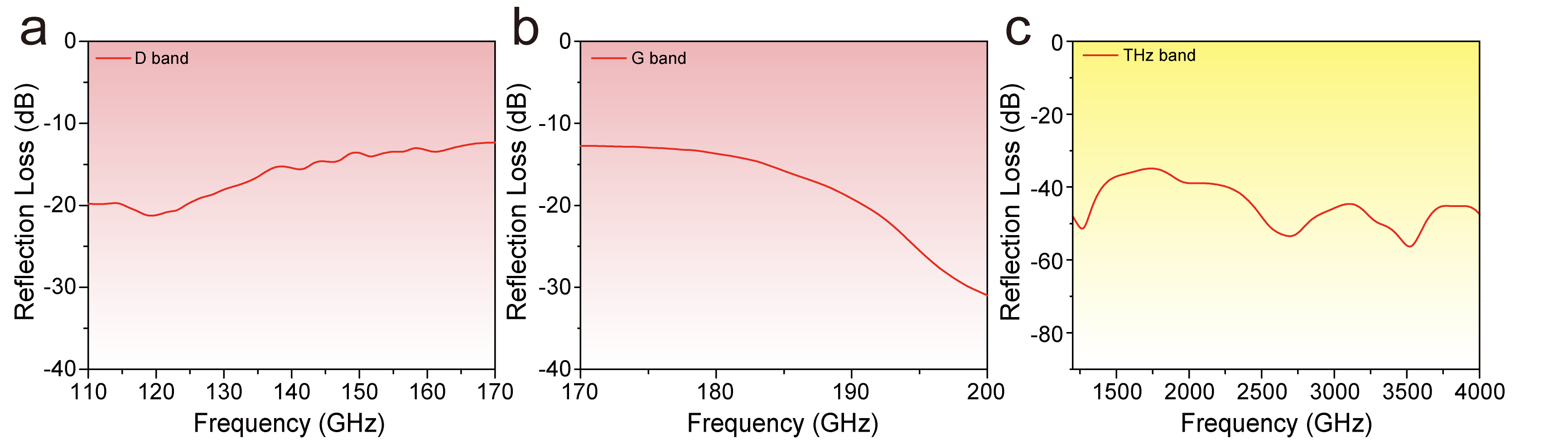


**Figure S17.** Supplementary RL curves of the G2C25H2 structure in the D/G bands (110–170 GHz, 170–220 GHz) and THz ranges: (a) 110–170 GHz, (b) 170–200 GHz, and (c) 1.2–4.0 THz.


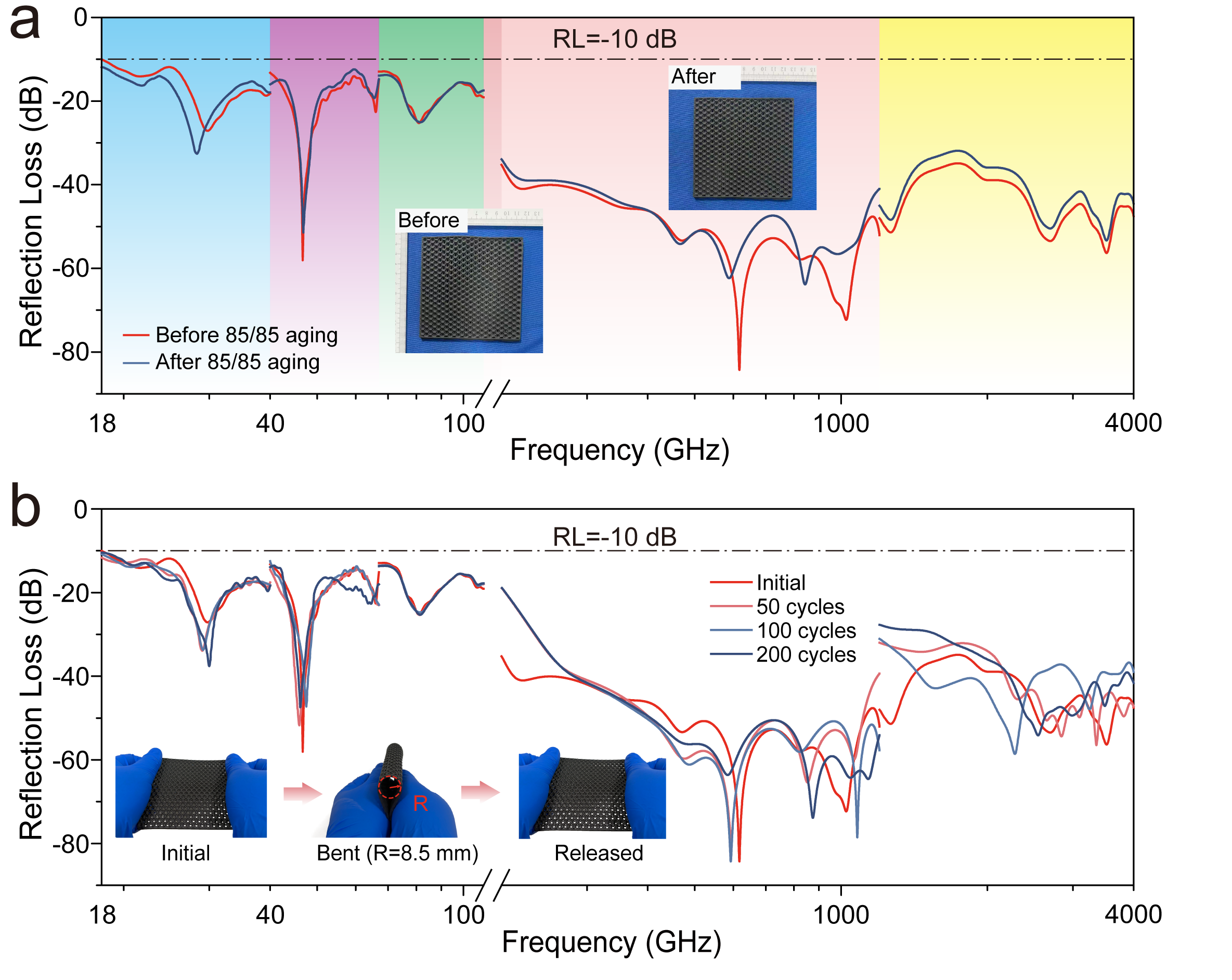


**Figure S18.** Broadband electromagnetic stability of the GCH absorber after durability tests. (a) RL curves before and after 85 °C/85% RH aging for 168 h. (b) RL curves after repeated bending-releasing cycles at a bending radius (R) of 8.5 mm.


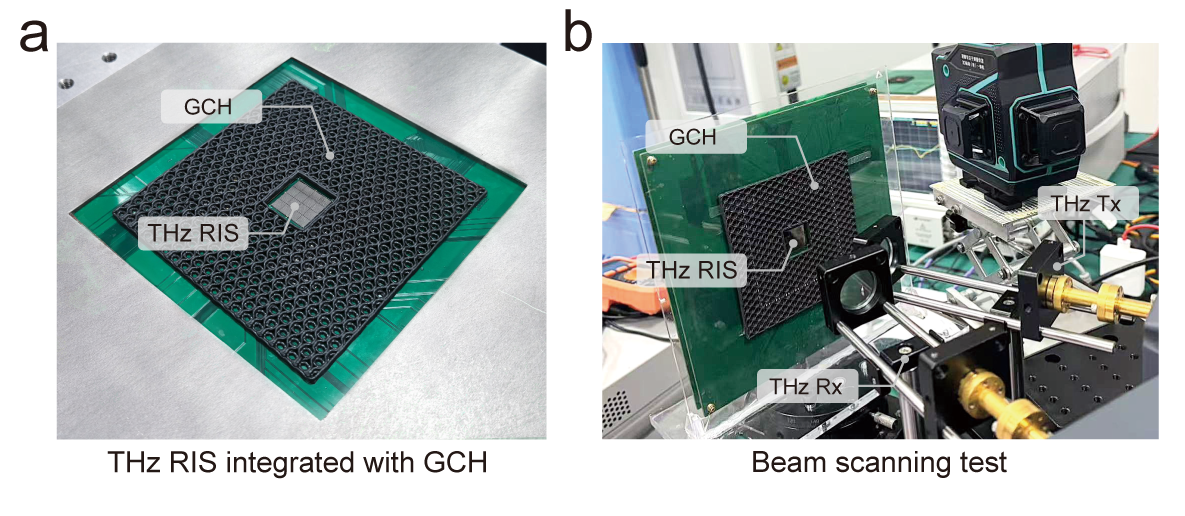


**Figure S19.** (a) Optical photograph of the fabricated THz RIS integrated with the 3D-printed GCH absorbing structure. (b) Photograph of the THz beam scanning test setup for the integrated RIS device.


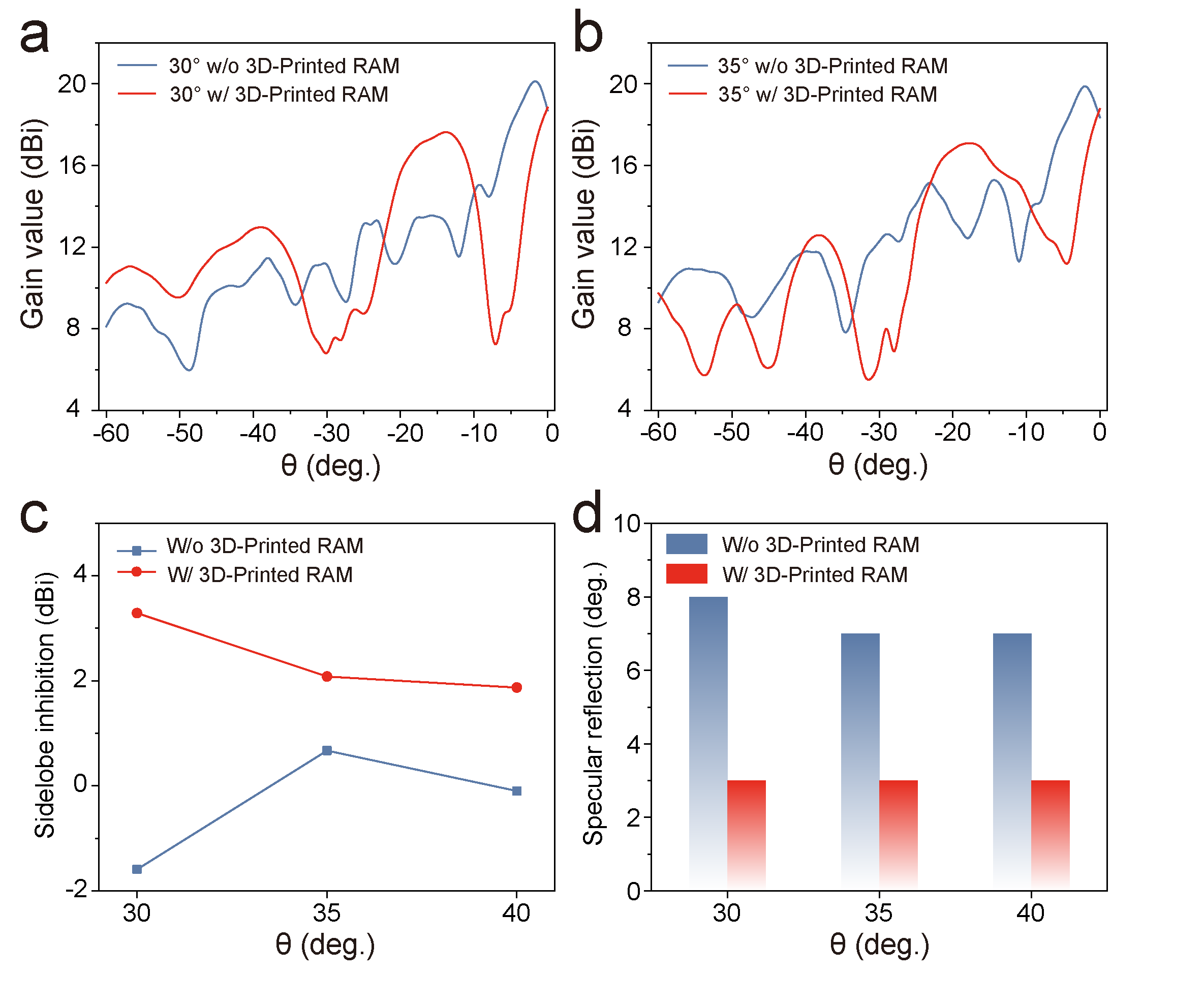


**Figure S20.** (a)–(b) Comparison of antenna gain with scanning angle θ before and after introducing 3D printed RAM at 220 GHz under scanning angles of 30° and 35°, respectively. (c) Quantitative comparison of sidelobe suppression levels at different scanning angles. (d) Statistical results of specular reflection beamwidth.


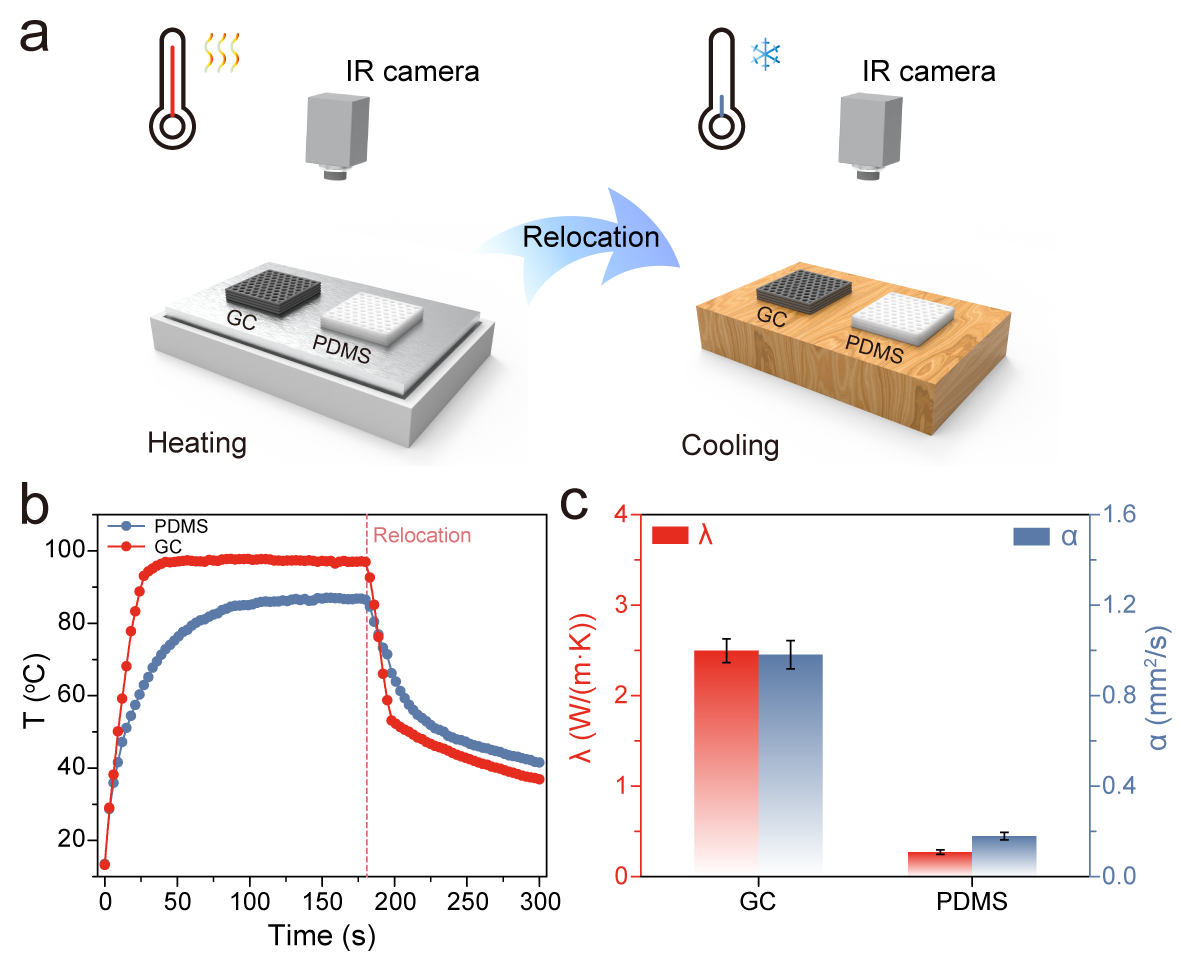


**Figure S21.** Thermal conductivity and surface heat-transfer behavior of GC composite and pristine PDMS. (a) Schematic illustration of the infrared thermal-imaging test. The samples were first placed on a 100 °C hot stage for heating and then rapidly transferred to a room-temperature wooden substrate for cooling. (b) Surface temperature evolution of GC and PDMS during the heating–cooling process. (c) Thermal conductivity and thermal diffusivity of GC and PDMS measured at 70 °C.


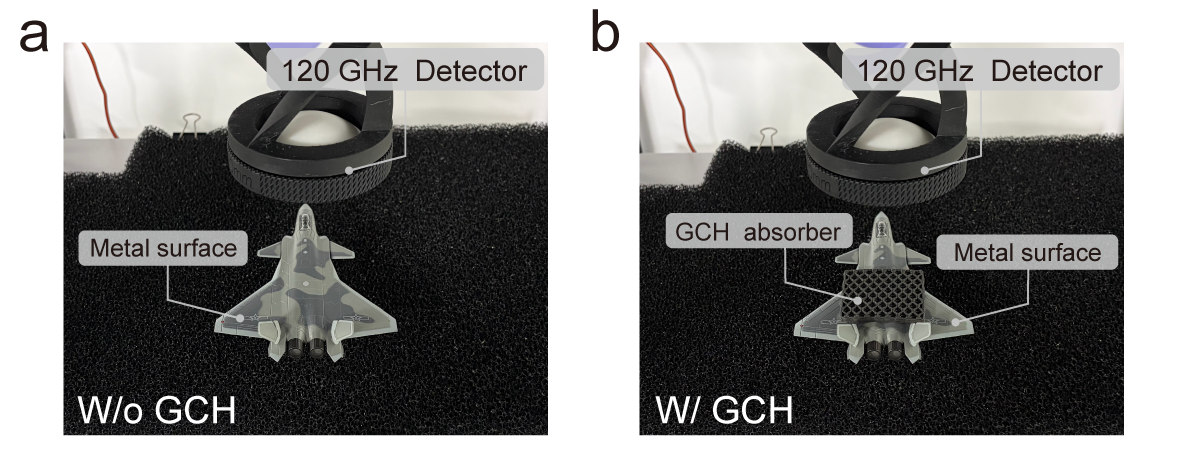


**Figure S22.** Images illustrate the fighter jet model both without (a) and with (b) the GCH absorber affixed to its fuselage.

| **Component**  **Number** | **Gr/g** | **CIP/g** | **PDMS/g** | **Curing agent/g** |
| --- | --- | --- | --- | --- |
| G_0_C_50_ | 0.0 | 50.0 | 10.0 | 1.0 |
| G_1_C_37.5_ | 1.0 | 37.5 | 10.0 | 1.0 |
| G_2_C_25_ | 2.0 | 25.0 | 10.0 | 1.0 |
| G_3_C_12.5_ | 3.0 | 12.5 | 10.0 | 1.0 |
| G_4_C_0_ | 4.0 | 0.0 | 10.0 | 1.0 |

**Table S1.** The detailed compositions of GC functional inks.

**Table S2.** The detailed printing information.

| **X/Y Axis**  **movement speed (mm/s)** | **Z Axis**  **movement speed (mm/s)** | **X/Y axis movable limit**  **(mm)** | **Z axis movable limit**  **(mm)** | **Syringe volume**  **(mL)** |
| --- | --- | --- | --- | --- |
| 15 | 5 | 300 | 40 | 50 |

**Table S3.** Comparison with previously reported absorbing materials.

| **Materials** | **Technique** | **Thickness**  **(mm)** | **EAB**  **(GHz)** | **RL_min_**  **(dB)** | **EAB/t**  **(GHz·mm^-1^)** | **Ref** |
| --- | --- | --- | --- | --- | --- | --- |
| mSiO_2_ | Coating | 1.8 mm | 2300 GHz (0.2-2.5 THz) | -29.8 | 1277.77 | [S1] |
| CIP/MXene | Coating | 1.5 mm | 2973.5 GHz (26.5~3000 GHz) | -27.9 | 1486.75 | [S2] |
| 3D Graphene | Foam | 4mm | 1045 GHz (0.155-1.2 THz) | -19.0 | 261.25 | [S3] |
| Si_3_N_4_–SiC | Foam | 2.80 | 8.78 GHz (9.20~17.98 GHz) | -51.6 | 3.16 | [S4] |
| MXene/GO | Foam | 4.00 | 1200 GHz (0.2~2.0 THz) | -37.0 | 300.00 | [S5] |
| Gr/CF | Aerogel | 3.25 | 1208.72 GHz (9.28-18 GHz/ 0.3-1.5 THz) | -64.8 | 371.91 | [S6] |
| Gr/MWCNT | Aerogel | 8.00 | 25.7 GHz  (5.8~18 GHz/ 26.5~40 GHz) | -39.7 | 3.21 | [S7] |
| Ag-Cu alloy | 3D-printing | 20.00 | 37 GHz (3~40 GHz) | -24.5 | 1.85 | [S8] |
| RGO/CIP | 3D-printing | 3.20 | 3008.24 GHz (8.99-17.23GHz/ 0.5-3.5 THz) | -52.2 | 940.07 | [S9] |
| RGO | 3D printing | 6.40 | 35.5 GHz (4.5-40 GHz) | -35.5 | 5.55 | [S10] |
| CIP/Gr | 3D printing | 4.25 | 3982 GHz  (18~4000 GHz) | -84.3 | **936.94** | **This Work** |
| CIP/Gr | 3D printing | 2.60 | 3933 GHz  (67~4000 GHz) | -84.3 | **1512.69** | **This Work** |

(GF: Glass fiber, CF: Carbon Fiber, RGO: Reduced graphene oxide, Gr: Graphene, CNT: Carbon nanotube, MWCNT: Multi-walled carbon nanotube, CIP: Carbonyl iron powder, MXene: Ti_3_C_2_T_x_).

**Supplementary Reference**

1. Y. Guo, Z. Wang, S. Bi, Q. Sun, Y. Lu. Design and regulation of electromagnetic parameters of thz absorbing epoxy resin composite film for 6G electronic packaging. Mater. Today Phys. **51**, 101655 (2025). <https://doi.org/10.1016/j.mtphys.2025.101655>
2. Z. Chen, M. Luo, W. Jiao, Y. Jiang, J. Xie, et al. Multiscale magnetic-electric synergy in CIP/MXene/epoxy nano-micro composites for ultra-broadband absorption and enhanced thermal conductivity. Compos. Sci. Technol. **271**, 111360 (2025). <https://doi.org/10.1016/j.compscitech.2025.111360>
3. Z. Huang, H. Chen, Y. Huang, Z. Ge, Y. Zhou, et al. Ultra‐broadband wide‐angle terahertz absorption properties of 3D graphene foam. Adv. Funct. Mater. **28**(2), 1704363 (2017). <https://doi.org/10.1002/adfm.201704363>
4. Z. Xing, X. You, H. Ouyang, Q. Zhang, Y. Yang, et al. Porous and lightweight continuous sic fiber reinforced Si_3_N_4_–SiC composites for wide frequency electromagnetic wave absorption. Composites, Part B. **300**, 112497 (2025). <https://doi.org/10.1016/j.compositesb.2025.112497>
5. W. Ma, H. Chen, S. Hou, Z. Huang, Y. Huang, et al. Compressible highly stable 3d porous MXene/GO foam with a tunable high-performance stealth property in the terahertz band. ACS Appl. Mater. Interfaces. **11**(28), 25369-25377 (2019). <https://doi.org/10.1021/acsami.9b03406>
6. Y. Cao, Z. Cheng, R. Wang, X. Liu, T. Zhang, et al. Multifunctional graphene/carbon fiber aerogels toward compatible electromagnetic wave absorption and shielding in gigahertz and terahertz bands with optimized radar cross section. Carbon. **199**, 333-346 (2022). <https://doi.org/10.1016/j.carbon.2022.07.077>
7. Q. Zhang, Z. Du, M. Hou, Z. Ding, X. Huang, et al. Ultralight, anisotropic, and self-supported graphene/MWCNT aerogel with high-performance microwave absorption. Carbon. **188**, 442-452 (2022). <https://doi.org/10.1016/j.carbon.2021.11.047>
8. Z. Yang, Q. Liang, Y. Duan, P. Liu, X. Wang, D. Li. Electromagnetic characteristics and 3D-printing realization of a lightweight hierarchical wave-absorbing metastructure for low-frequency broadband absorption. J. Alloys Compd. **949**, 169894 (2023). <https://doi.org/10.1016/j.jallcom.2023.169894>
9. P. Liu, S. Shi, Y. Ni, K. Xu, Q. Gao, et al. Direct ink writing printed flexible double-layer staggered woodpile structure for multi-band compatible absorption of gigahertz and terahertz waves. Chem. Eng. J. **478**, 147474 (2023). <https://doi.org/10.1016/j.cej.2023.147474>
10. L. Yin, X. Tian, Z. Shang, D. Li. Ultra-broadband metamaterial absorber with graphene composites fabricated by 3D printing. Mater. Lett. **239**, 132-135 (2019). <https://doi.org/10.1016/j.matlet.2018.12.087>
